# Antibiotic-induced perturbations in microbial diversity during post-natal development alters amyloid pathology in an aged APP_SWE_/PS1_ΔE9_ murine model of Alzheimer’s disease

**DOI:** 10.1038/s41598-017-11047-w

**Published:** 2017-09-05

**Authors:** Myles R. Minter, Reinhard Hinterleitner, Marlies Meisel, Can Zhang, Vanessa Leone, Xiaoqiong Zhang, Paul Oyler-Castrillo, Xulun Zhang, Mark W. Musch, Xunuo Shen, Bana Jabri, Eugene B. Chang, Rudolph E. Tanzi, Sangram S. Sisodia

**Affiliations:** 1 Department of Neurobiology, The University of Chicago, Chicago, IL 60637 USA; 2 The Microbiome Center, The University of Chicago, Chicago, IL 60637 USA; 3 Department of Medicine, The University of Chicago, Chicago, IL 60637 USA; 4 Committee on Immunology, The University of Chicago, Chicago, IL 60637 USA; 5 Department of Neurology, Genetics and Aging Research Unit, MassGeneral Institute for Neurodegenerative Diseases, Massachusetts General Hospital, Charlestown, MA 02114 USA

## Abstract

Recent evidence suggests the commensal microbiome regulates host immunity and influences brain function; findings that have ramifications for neurodegenerative diseases. In the context of Alzheimer’s disease (AD), we previously reported that perturbations in microbial diversity induced by life-long combinatorial antibiotic (ABX) selection pressure in the APP_SWE_/PS1_ΔE9_ mouse model of amyloidosis is commensurate with reductions in amyloid-β (Aβ) plaque pathology and plaque-localised gliosis. Considering microbiota-host interactions, specifically during early post-natal development, are critical for immune- and neuro-development we now examine the impact of microbial community perturbations induced by acute ABX exposure exclusively during this period in APP_SWE_/PS1_ΔE9_ mice. We show that early post-natal (P) ABX treatment (P14-P21) results in long-term alterations of gut microbial genera (predominantly *Lachnospiraceae* and *S24-7*) and reduction in brain Aβ deposition in aged APP_SWE_/PS1_ΔE9_ mice. These mice exhibit elevated levels of blood- and brain-resident Foxp3^+^ T-regulatory cells and display an alteration in the inflammatory milieu of the serum and cerebrospinal fluid. Finally, we confirm that plaque-localised microglia and astrocytes are reduced in ABX-exposed mice. These findings suggest that ABX-induced microbial diversity perturbations during post-natal stages of development coincide with altered host immunity mechanisms and amyloidosis in a murine model of AD.

## Introduction

Alzheimer’s disease (AD) is a chronic neurodegenerative disorder that is pathologically characterised by the extracellular deposition of amyloid-β (Aβ) peptides in senile plaques and the intracellular accumulation of hyper-phosphorylated forms of tau protein in neurofibrillary tangles. In addition, positron emission tomography has revealed that plaque-localised gliosis and neuro-inflammation is consistently observed and is highly correlated with cognitive decline^1, 2^. Combined with data from recent genome-wide association studies identifying numerous polymorphisms within genes critical for innate immune responses (i.e., *CD33* and *TREM-2* ^3–6^) that confer elevated risk of late onset AD, it has been widely speculated that a deleterious neuro-inflammatory response may be required to promote Aβ-induced cognitive decline in AD^7–9^.

As microglia express high levels of pattern recognition receptors that detect soluble Aβ peptides leading to robust inflammasome activation^8^, these cell types are considered the primary responders to Aβ deposition in the brain and initiate neuro-inflammation. Importantly, microglia can display a myriad of heterogeneous pro- or anti-inflammatory activation states that remain dependent on the initial stimulus and inflammatory micro-environment^10, 11^. In this regard, efforts to identify specific microglial phenotypes and activation mechanisms responsible for enhanced Aβ phagocytosis and clearance in AD have been inconclusive^12^.

It should be noted that the neuro-inflammatory response is not solely restricted to contributions by resident microglia, astrocytes and neurons of the brain, as infiltrating peripheral T cell compartments are known to propagate neuro-inflammation in preclinical models of AD^13^. While previous research investigating T-regulatory cell (T-reg) activity in facets of CNS repair remains conflicting^14–17^, numerous studies suggest a beneficial, albeit complex, role of T-reg function in mitigating AD-like disease pathology. In this regard, IL-2-mediated activation of the proliferative capacity of Foxp3^+^ T-regs alleviates cognitive deficits in the Thy1-APP_SWE_/PS1_L166P_ mouse model of Aβ amyloidosis^18^ and adoptive transfer of these cells improves spatial and temporal learning and reduces cortical and hippocampal Aβ plaque deposition in 3xTg familial AD (FAD) mice^19^. Furthermore, transient depletion, and subsequent re-population, of Foxp3^+^ T-regs is sufficient to re-balance neuro-inflammatory responses by increasing T-reg and macrophage recruitment to the CNS and reduces amyloid deposition in the accelerated 5xFAD mouse model of AD^20^. Finally, antibody-mediated blockade of the PD-1 immune checkpoint in multiple APP/PS1 transgenic AD models triggers elevated IFNγ^+^ myeloid cell recruitment to the central nervous system (CNS), reductions in amyloid pathology and alleviates cognitive deficits^21^. These studies highlight the capacity of T-reg populations to circulate from peripheral tissues to the CNS upon an immune system “re-balance”, with these recruited cells capable of alleviating AD-like pathology provided the inflammatory niche is permissive. While there is a growing body of evidence indicating an important role for peripheral and central immunity regulating neuro-inflammatory responses in mouse models^22^, the mechanisms responsible for initiating these immune responses and coordinating this interplay remain largely unknown.

The commensal microbiome, the vast populations of micro-organisms and their immunologically-active metabolites that colonise the host, continues to emerge as a significant entity shaping homeostatic control of host immunity^23^. The microbiome closely regulates the innate lymphoid cell populations of the gastro-intestinal (GI) tract^24^ and these cells can, in turn, modulate T-cell populations that reside in the mesenteric lymph nodes (MLNs)^25^ that have the capacity to circulate and enter the brain by transversing the choroid plexus^26^. The microbially-derived short chain fatty acid (SCFA) butyrate can induce differentiation and extra-thymic generation of peripherally-localised T-regs^27, 28^, sharing this same periphery-CNS-homing capacity. Additionally, the microbiome also imparts direct effects on microglial development as germ-free (GF) mice, exhibiting a complete lack of microbial colonisation, display remarkably altered microglial transcriptomic profiles controlling cellular surveillance and response to bacterial or viral challenge^29^. Supplementation of GF mice with SCFAs reverts these alterations to exhibit microglial phenotypes akin to specific pathogen free (SPF)-housed mice^29^ and highlights the importance of not just the microbiota themselves, but their metabolic products, in regulating facets of brain function.

Extending the notion of the microbial influence on brain physiology, emerging evidence now suggests that perturbations in microbial diversity influences the pathology of many neurodegenerative disorders^30, 31^. Mice supplemented with broad-spectrum antibiotics (ABX), to deplete microbiota, and subjected to the middle cerebral artery occlusion model of ischemic stroke displayed elevated levels of circulating Foxp3^+^ T-regs, reduced brain infract volume and improved sensorimotor function^32^. GF transgenic mice that overexpress α-synuclein (α-syn), the primary pathogenic peptide in Parkinson’s disease (PD), display improved motor function and attenuated α-syn brain aggregation compared to SPF-housed mice^33^. In clinically diagnosed AD patients, abundance of specific pro-inflammatory gut bacteria taxa, including *Escherichia* and *Shigella*, has been positively correlated with pro-inflammatory cytokine expression^34^. Indicating a prominent role of microbial signals during AD pathogenesis, we previously reported that ABX treatment of male APP_SWE_/PS1_ΔE9_ mice over 6 months induces perturbations in gut microbial diversity alongside alterations in peripherally circulating inflammatory cytokines and chemokines that are correlated with an attenuation of Aβ deposition and plaque-localised glial reactivity^35^. Supporting these findings, a recent study demonstrated that GF Thy1-APP_SWE_/PS1_L166P_ mice exhibit dampened neuro-inflammatory responses and reductions in brain and blood Aβ peptide burden^36^. These studies support a complex role for the commensal microbiota in regulating host immunity parameters and neurodegenerative pathology in multiple disease models.

A vast body of evidence now suggests the post-natal developmental time-frame, particularly during the post-weaning phase, is where the commensal microbiome is most susceptible to prolonged perturbation^37–39^ and represents a crucial developmental window^40^ by which microbiota-host interactions mediate immuno- and neuro-development that may impact host physiology in later life^41–45^. Considering the aforementioned GF and ABX-treated APP/PS1 mouse studies wherein microbes are perturbed throughout the entirety of life^35, 36^, we aimed to investigate the effect of microbial perturbations during the crucial post-natal developmental window alone in the APP_SWE_/PS1_ΔE9_ model. In the current study, we tested the hypothesis that ABX-induced perturbation in microbial diversity exclusively during early post-natal development of APP_SWE_/PS1_ΔE9_ mice would be sufficient to recapitulate the phenotypic alterations observed in our earlier study^35^. We observe that APP_SWE_/PS1_ΔE9_ mice, gavaged daily with high-dose ABX between P14-P21 (1 wk ABX gvg), but raised thereafter under standard SPF conditions, exhibit significant alterations in the abundance of specific gut bacterial taxa and elevated blood-circulating and brain-localised Foxp3^+^ T-regs that correlate with an altered inflammatory profile and attenuated Aβ plaque deposition in aged mice. In addition, we observed reductions in plaque-associated microglia and astrocytes. Our findings suggest that in this murine model of Aβ amyloidosis, short-term ABX treatment-induced perturbations in the commensal microbial composition at an early period of host post-natal development has a significant impact on host immunity and brain Aβ plaque deposition in later life.

## Results

### ABX treatment during post-natal development induces specific alterations in gut microbial diversity in aged APP_SWE_/PS1_ΔE9_ mice

**Figure 1:**
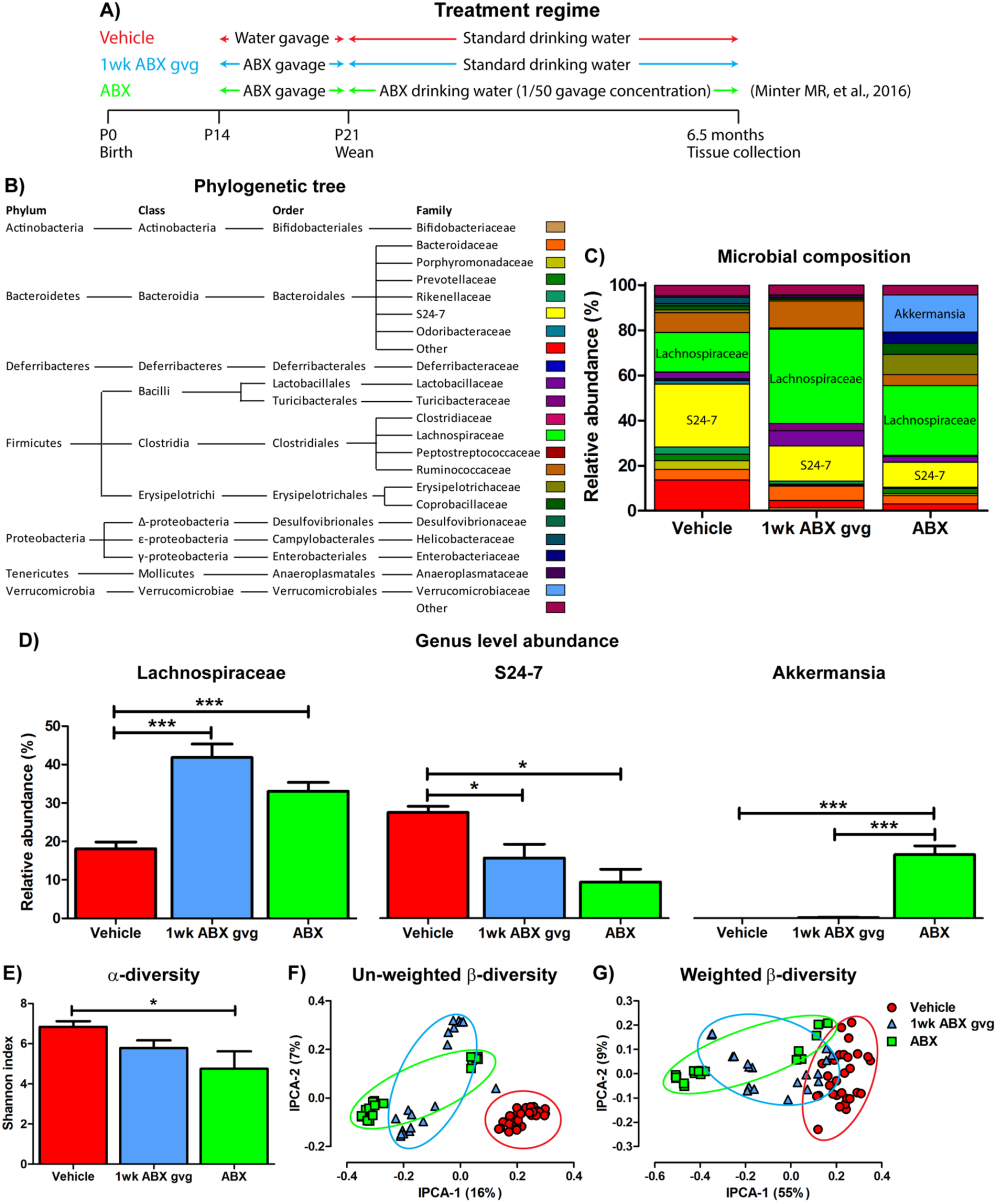
Alterations in gut microbial diversity induced by 1 wk post-natal ABX gvg treatment of aged APP_SWE_/PS1_ΔE9_ mice. (**A**) Treatment regime schematic detailing the antibiotic (ABX) treatment of APP_SWE_/PS1_ΔE9_ mice used in the current and previous study^35^. (**B**) Phylogenetic tree describing taxonomy assignment of the (**C**) bacterial diversity histogram generated from Illumina® MiSeq based V4-V5 amplicon sequencing of the 16 s rRNA gene from caecal and faecal contents of 6.5 month old male APP_SWE_/PS1_ΔE9_ mice. Only quality-controlled OTU reads corresponding to bacterial families with relative abundance >0.5% were included. (**D**) Relative abundance comparisons of *Lachnospiraceae*, *S24-7* and *Akkermansia*, the top three differentially expressed bacterial genus’ identified by V4-V5 amplicon sequencing of the 16 s rRNA gene (n = 10–12, *p < 0.05, ***p < 0.001, one-way ANOVA with Tukey’s multiple comparison post-hoc test). (**E**) Shannon index analysis of the V4-V5 amplicon 16 s rRNA gene sequencing as a measure of microbial α-diversity in vehicle, 1 wk ABX gvg and ABX-treated 6.5 month old APP_SWE_/PS1_ΔE9_ mice (*n* = 10–12, *p < 0.05, one-way ANOVA with Tukey’s multiple comparison post-hoc test). Unifrac principal co-ordinate analysis of (**F**) un-weighted, accounting for presence of OTUs only, and (**G**) weighted β-diversity, accounting for both presence and relative abundance of OTUs, in vehicle, 1 wk ABX gvg and ABX-treated APP_SWE_/PS1_ΔE9_ mice (*n* = 10–12 mice, caecal and faecal sequencing, ellipses represent treatment groupings). The percentage of data variance explained by each IPCA is displayed. Data are displayed as X/Y scatter, mean or mean ± SEM. All data from the ABX treatment group is reproduced from Minter M. R. *et al*.^35^ and is included here solely for sake of comparison. See Supp. Figs 1, 3, Supp. Table 1 and statistical Table 2 for additional information.

We previously reported that ABX supplementation throughout the lifespan of aged APP_SWE_/PS1_ΔE9_ mice leads to altered gut microbial diversity, attenuation of Aβ plaque deposition in the brain and alterations in levels of cytokines and chemokines associated with innate immunity^35^. Evidence suggests the post-natal development time-frame, particularly during the pre-weaning phase^40^, is a crucial window by which the commensal microbiome is susceptible to sustained perturbation and regulates multiple facets of host physiology^41–45^. To address if microbial diversity during this post-natal development window impacts on amyloidosis in later life, we investigated if daily gavage (gvg) with high-dose ABX only during the peri-weaning period, from P14-P21, might recapitulate the phenotypes observed previously in APP_SWE_/PS1_ΔE9_ mice subjected to life-long ABX exposure^35^. To assess the degree to which the 1 wk ABX gvg treatment (Fig. 1A) could affect microbial abundance and diversity at 6.5 months of age, the typical age-of-onset for amyloid deposition in APP_SWE_/PS1_ΔE9_ mice, we utilised 16 s rRNA gene Q-PCR (Supp. Fig. 3A,B), 16 s rRNA gene terminal restriction polymorphism analysis (T-RFLP, Supp. Fig. 3C,D) and Illumina® MiSeq amplicon sequencing of the V4-V5 variable region of the 16 s rRNA gene (Fig. 1B–G, Supp. Table 1) to analyse gut-residing microbiota.

Q-PCR analysis of 16 s rRNA gene copy number in total DNA isolated from caecal (Supp. Fig. 3A) and faecal matter (Supp. Fig. 3B) revealed no statistical difference in bacterial abundance between vehicle and 1 wk ABX gvg-treated APP_SWE_/PS1_ΔE9_ mice at the time of cull. This observation was apparent despite the strong efficacy of ABX treatment in preventing cultivable bacteria growth from faecal matter obtained immediately post-1 wk gvg (Supp. Fig. 1B,C). T-RFLP analysis of the amplified 16 s rRNA gene from caecal (*n* = 10-13, Supp. Fig. 3C) and faecal contents (*n* = 8-9, Supp. Fig. 3D) with subsequent principal coordinate analysis (PCA) plot generation reveals distinct stratification of vehicle and 1 wk ABX gvg APP_SWE_/PS1_ΔE9_ treatment groups. Collectively these findings imply that while the gut microbiota recovers to steady-state abundance levels in aged APP_SWE_/PS1_ΔE9_ mice post-ABX gvg, the microbial composition is likely altered.

To further classify differential microbial profiles in vehicle and 1 wk ABX gvg-treated APP_SWE_/PS1_ΔE9_ mice, we performed Illumina® MiSeq amplicon sequencing of the V4-V5 variable region of the 16 s rRNA gene. Additionally, we compared the vehicle and 1 wk ABX gvg treatment groups to our previously reported sequencing data of APP_SWE_/PS1_ΔE9_ mice that received ABX throughout the entire lifespan (ABX group)^35^. Histogram plots of microbial composition identified by operational taxonomic unit (OTU) read alignment and stratification against a bacterial phylogenetic tree (Fig. 1B) reveals alterations in gut microbial composition, at the family level, in both ABX treatment groups compared to vehicle controls (Fig. 1C). Relative abundance analysis of three significantly altered bacterial classes at the genus level revealed significant expansion of *Lachnospiraceae* (Vehicle: 18.09 ± 1.76% vs. 1 wk ABX gvg: 41.84 ± 3.53% vs. ABX: 33.07 ± 2.30%, *n* = 10–12, p < 0.001), reduction of *S24-7* (Vehicle: 27.51 ± 1.64% vs. 1 wk ABX gvg: 15.70 ± 3.79% vs. ABX: 9.42 ± 3.33%, *n* = 10–12, p < 0.05) and expansion of *Akkermansia* (Vehicle: 0.02 ± 0.002%; vs. 1 wk ABX gvg: 0.19 ± 0.066%; vs. ABX: 16.51 ± 2.31% vs., *n* = 10–12, p < 0.001) between both ABX treatment regimens and vehicle controls (Fig. 1D).

To confirm our initial T-RFLP microbial diversity analysis, we investigated α-diversity by calculating Shannon indices that assume all microbial species are represented in our samples and that random sampling occurs. Using this index, we observed a non-statistically significant decrease in microbial α-diversity in 1 wk ABX gvg-treated APP_SWE_/PS1_ΔE9_ mice compared to vehicle controls, and this was not as prominent as previously observed in the ABX group (Vehicle: 6.94 ± 0.28 vs. 1 wk ABX gvg: 5.78 ± 0.38 vs. ABX: 4.75 ± 0.87, p < 0.05, *n* = 10–12, Fig. 1E)^35^. We then assessed the dissimilarity of individual microbial communities between treatment groups (β-diversity). PCA plotting of un-weighted β-diversity dissimilarity matrix data, accounting for presence of OTUs but not abundance, revealed a distinct clustering effect in both ABX treatment regimens of APP_SWE_/PS1_ΔE9_ mice compared to vehicle controls (*n* = 10–12, individual faecal and caecal reads, Fig. 1F). Examination of weighted β-diversity PCA dissimilarity matrix data, accounting for presence and abundance of OTUs, also revealed a similar distinct clustering effect in both ABX treatment regimens of APP_SWE_/PS1_ΔE9_ mice compared to vehicle controls (*n* = 10–12, individual faecal and caecal reads, Fig. 1G).

We acknowledge that there appears to be differential clustering in β-diversity in our ABX-treated groups. Upon closer analysis, we determined that the origin of this differential clustering could be traced back to individual litters, that were housed in separate cages. This raises the possibility of a ‘cage/litter’ effect but we believe these studies were significantly powered enough to overcome these potential caveats. Additionally, we re-analysed data of these mice pertinent to amyloid load and microglial/astrocytic cell counts (see below) and found these data points not to fall outside of the 2.5*interquartile range threshold for determining outliers.

Collectively these data highlight that microbial diversity, but not total abundance, is altered by either post-natal or lifetime ABX treatment at the time of cull in APP_SWE_/PS1_ΔE9_ mice. It is important to note that the 1 wk ABX gvg treatment regimen employed in the current study induces a milder diversity perturbation, evidenced by α and β-diversity analysis, in GI-resident microbiota compared to the previously observed life-long ABX treatment of APP_SWE_/PS1_ΔE9_ mice^35^. In contrast to our previous observations of the enlarged cecum of life-long ABX-treated animals^35^, we did not observe any gross morphological alterations in the GI tract of 1 wk ABX gvg-treated APP_SWE_/PS1_ΔE9_ mice (Supp. Fig. 1D,E).

### Peripheral and central immune cell compartments in aged APP_SWE_/PS1_ΔE9_ mice are altered by early post-natal ABX treatment

Recent studies suggest an important role of T-cell activity, specifically Foxp3^+^ T-regs in coordinating brain neuro-inflammatory responses and regulating neuropathology observed in preclinical models of AD^18, 20, 21, 46^. Emerging evidence strongly supports a role of the commensal microbiota, and their metabolites, in regulating innate lymphoid cell populations that in turn influence lymph node-residing and blood-circulating T-cell phenotypes that traverse the choroid plexus and infiltrate the brain parenchyma^24–28^. Hence, we isolated mononuclear cells from MLNs, whole blood and brain of vehicle and 1 wk ABX gvg-treated APP_SWE_/PS1_ΔE9_ mice and analysed T-lymphocyte populations by flow cytometry.

**Figure 2:**
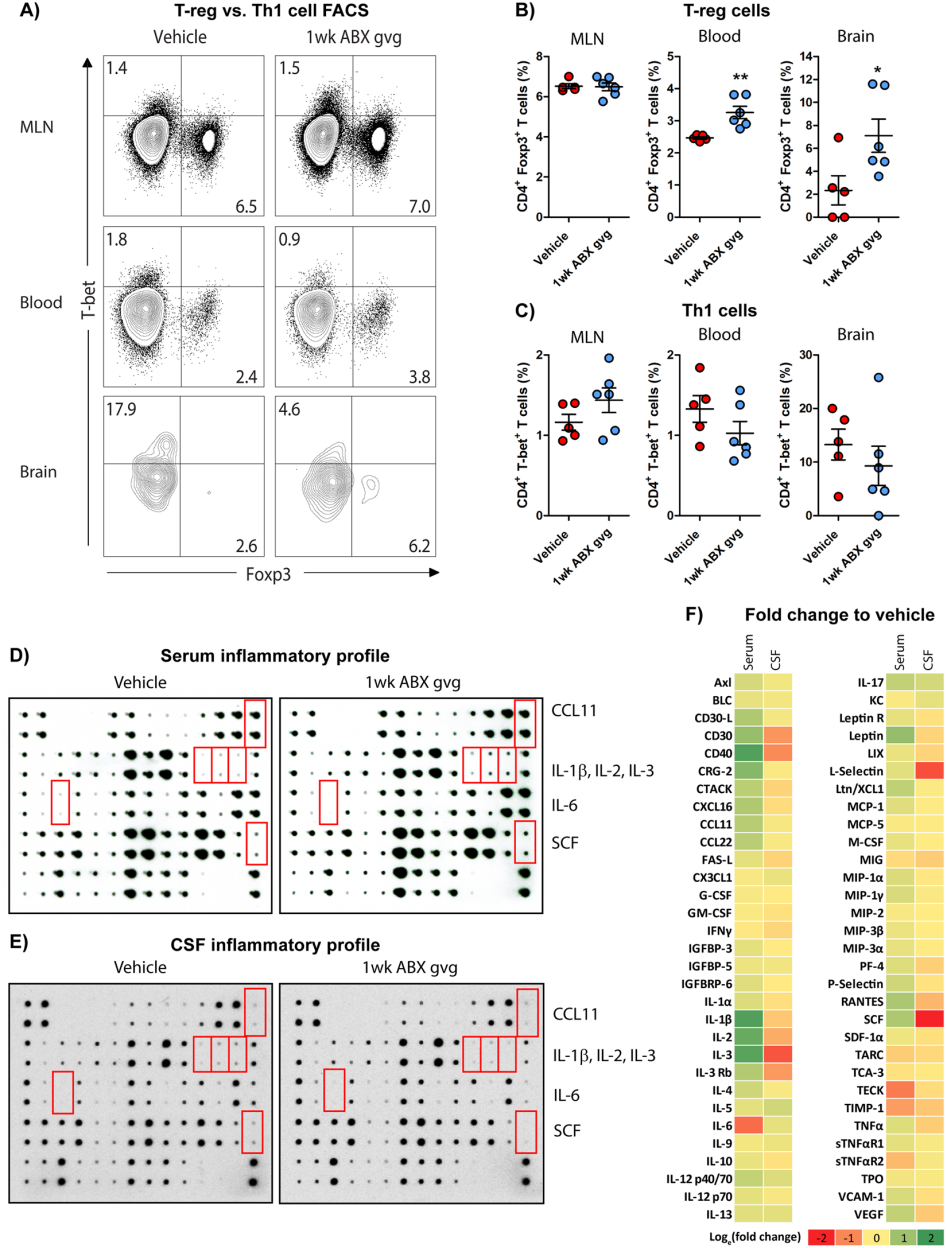
1 wk ABX gvg-treated APP_SWE_/PS1_ΔE9_ mice display altered peripheral and brain inflammatory profiles. (**A**) Representative density dot plots of T-bet and Foxp3 intracellular expression in TCRβ^+^ CD4^+^ T cell populations isolated from MLN, blood and brain tissue of vehicle and 1 wk ABX gvg-treated APP_SWE_/PS1_ΔE9_ mice analysed by flow cytometry. Quantified percentages of (**B**) Foxp3^+^ and (**C**) T-bet^+^ CD4^+^ T cells, representative of a T-reg and Th1 T cell phenotype respectively, are expressed relative to total live CD4^+^ T cell counts (*n* = 5–6, *p < 0.05, **p < 0.01, un-paired two-tailed Student’s *t*-test). (**D**) Immunoblot-based array of inflammatory mediators present in the serum of vehicle and 1 wk ABX gvg-treated APP_SWE_/PS1_ΔE9_ mice (*n* = 10 pooled sera). (**E**) Immunoblot-based array of inflammatory mediators present in the CSF of vehicle and 1 wk ABX gvg-treated APP_SWE_/PS1_ΔE9_ mice (*n* = 10 pooled CSF). (**F**) Heat map analysis of inflammatory mediator fold change expression in 1 wk ABX gvg-treated APP_SWE_/PS1_ΔE9_ mice relative to control. Data are displayed as log_e_(mean) or mean ± SEM. See Supp. Figs 2, 4, 5, 6, and statistical Table 2 for additional information.

Assessment of Foxp3 and T-bet transcription factor expression levels (Fig. 2A, Supp. Fig. 2), representative of T-reg and Th1 populations respectively, revealed significant elevations in Foxp3^+^ T-regs (Vehicle (blood): 2.49 ± 0.04% vs. 1 wk ABX gvg (blood): 3.26 ± 0.19% **p = 0.0046; Vehicle (brain): 2.34 ± 1.27% vs. 1 wk ABX gvg (brain): 7.10 ± 1.45%, *p = 0.0387, *n* = 5–6, Fig. 2B) and non-statistically significant decreases in T-bet^+^ Th1 cell populations (Vehicle (blood): 1.33 ± 0.17% vs. 1 wk ABX gvg: 1.03 ± 0.15%; Vehicle (brain): 13.27 ± 2.88% vs. 1 wk ABX gvg (brain): 9.30 ± 3.67%, *n* = 5–6, Fig. 2C) in the blood and brain of 1 wk ABX gvg-treated APP_SWE_/PS1_ΔE9_ mice compared to vehicle controls. Analysis of the intracellular expression levels of GATA-3 and RorγT transcription factors, selective for Th2-like and Th17 lymphocyte populations, respectively, did not reveal any statistically significant differences in any of the tissues assessed between the two groups (Supp. Fig. 4A–F). In brain tissues of 1 wk ABX gvg-treated APP_SWE_/PS1_ΔE9_ mice, we noted non-statistically significant decreases in Th2-like responses (Vehicle (GATA-3): 2.46 ± 1.54% vs. 1 wk ABX gvg (GATA-3): 0.62 ± 0.62%, *n* = 5–6, Supp. Fig. 4C) and increased Th17 cell responses (Vehicle (RorγT): Undetectable vs. 1 wk ABX gvg (RorγT): 1.73 ± 0.64%, *n* = 5–6, Supp. Fig. 4F) but due to minimal detectable total GATA-3^+^ and RorγT^+^ cell counts in these samples, our data were highly variable. We also stimulated isolated mononuclear cell preparations with PMA/ionomycin to induce cytokine production and performed subsequent flow cytometry analysis. No statistically significant differences in abundance of CD4^+^ and CD8^+^ T cells that express IFNγ (Supp. Fig. 4G–L) or TNFα (Supp. Fig. 4M–R), and, CD4^+^ T cells expressing IL-4 (Supp. Fig. 4S–U) or IL-17 (Supp. Fig. 4V–X) were detected between vehicle and 1 wk ABX gvg-treated APP_SWE_/PS1_ΔE9_ mouse tissues. On the other hand, a statistically non-significant decrease in Th1-mediated cytokine expression was observed upon analysis of CD4^+^ T cells expressing IFNγ (Vehicle: 2.71 ± 0.83% vs. 1 wk ABX gvg: 1.18 ± 0.25%, *n* = 5–6, Supp. Fig. 4H) and TNFα (Vehicle: 46.39 ± 2.44% vs. 1 wk ABX gvg: 39.58 ± 2.32%, *n* = 5–6, Supp. Fig. 4N) in the blood of 1 wk ABX gvg-treated APP_SWE_/PS1_ΔE9_ mice. Collectively these data support the notion that APP_SWE_/PS1_ΔE9_ mice treated with ABX exclusively during post-natal development display enhanced numbers of circulating and brain-residing T-regs at the age of onset for brain amyloid deposition.

Additionally, we probed commercially available chemokine/cytokine arrays with serum and cerebrospinal fluid (CSF) isolated from vehicle and 1 wk ABX gvg-treated APP_SWE_/PS1_ΔE9_ mice to evaluate changes in the global inflammatory milieu (Fig. 2D–F). Densitometric analysis of these arrays revealed numerous changes in the inflammatory profile of pooled sera isolated from 1 wk ABX gvg-treated APP_SWE_/PS1_ΔE9_ mice compared to vehicle controls (*n* = 10 pooled serum, Supp. Fig. 5). Specifically, up-regulations of CCL11 (2.13-fold), IL-1β (6.12-fold), IL-2 (4.78-fold), IL-3 (5.33-fold) and stem cell factor (SCF, 2.35-fold) and down-regulations of IL-6 (0.28-fold) were identified in the 1 wk ABX gvg treatment group (Fig. 2F, Supp. Fig. 5). Densitometric analysis also revealed several alterations in the inflammatory mediator expression within the pooled CSF of 1 wk ABX gvg-treated APP_SWE_/PS1_ΔE9_ mice compared to vehicle controls (*n* = 10 pooled CSF, Supp. Fig. 5), albeit these changes were not to the similar magnitude compared to the serum results. Specifically, IL-2 (0.53-fold), IL-3 (0.22-fold) and SCF (0.02-fold) were downregulated in the 1 wk ABX gvg treatment group while CCL11, IL-1β and IL-6 expression remained unaltered (Fig. 2F, Supp. Fig. 5). These findings suggest that blood-circulating and CSF-residing inflammatory mediator levels are differentially altered in 1 wk ABX gvg-treated APP_SWE_/PS1_ΔE9_ mice and support the notion that developmental perturbations in microbial diversity may have long-term consequences for host innate immune responses.

**Figure 3:**
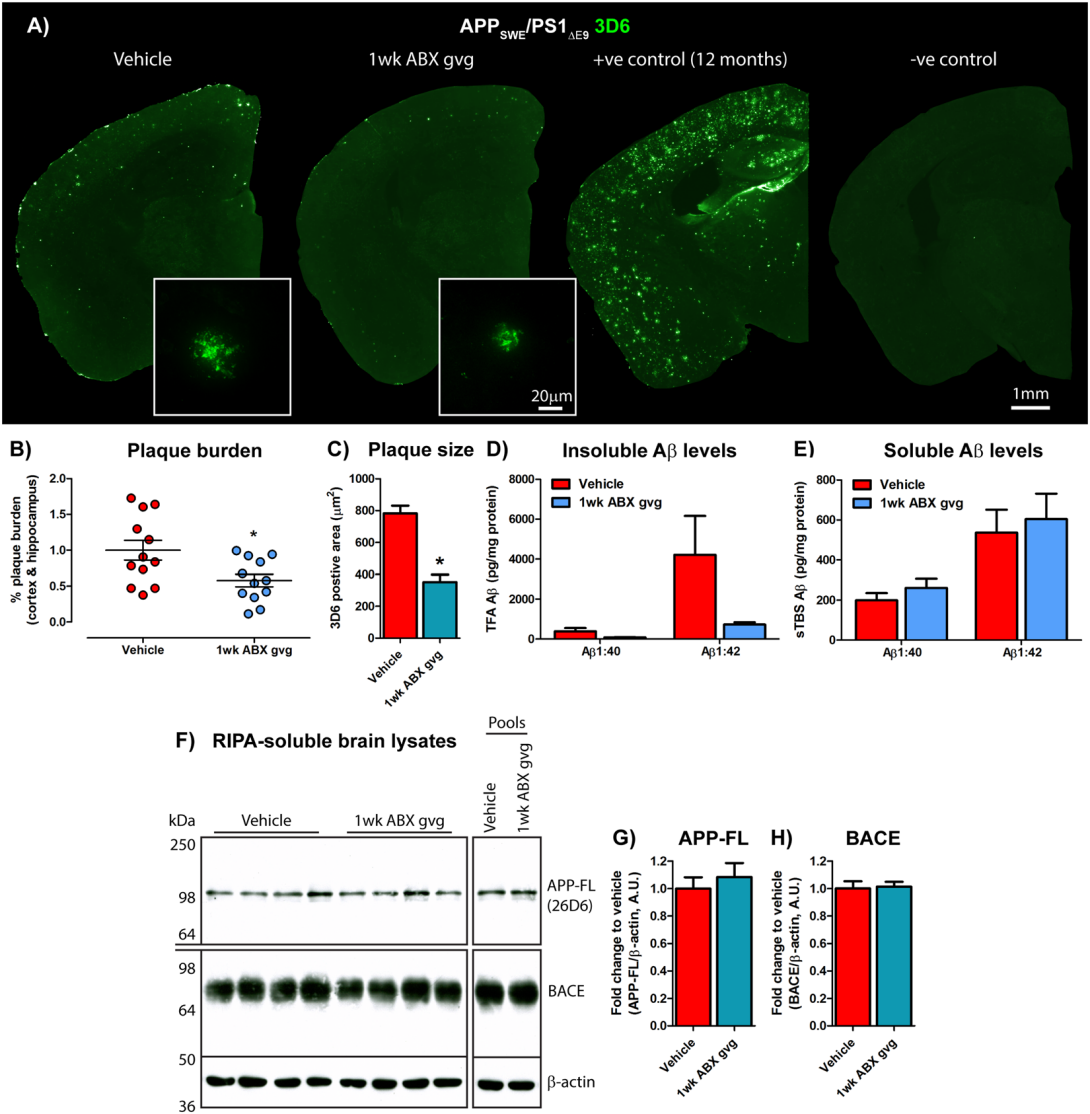
Amyloidosis is altered in 1 wk ABX gvg-treated APP_SWE_/PS1_ΔE9_ mice. (**A**) Representative immunohistochemical images of Aβ plaque burden in the cortex and hippocampus of vehicle and 1 wk ABX-gvg 6.5-month-old APP_SWE_/PS1_ΔE9_ mice using the anti-Aβ mAb, 3D6. Images in set display representative ×60 magnification z-stack maximum projections of individual 3D6^+^ Aβ plaques in these mice. Each immunohistochemical staining run was performed in conjunction with 12-month-old APP_SWE_/PS1_ΔE9_ mouse sections as a positive staining control and no primary antibody negative staining control. (**B**) Plaque burden quantification of vehicle and 1 wk ABX gvg-treated APP_SWE_/PS1_ΔE9_ mice using threshold-limited particle analysis of 3D6^+^ immunostaining (*n* = 12, *p < 0.05, un-paired two-tailed Student’s *t*-test). 3D6^+^ area was averaged from 6 sections/mouse (240 µm apart) and expressed relative to total cortical and hippocampal area of each slice. (**C**) Quantification of 3D6^+^ plaque area using threshold-limited immunofluorescence detection (*n* = 12, *p < 0.05, un-paired two-tailed Student’s *t*-test). (**D**) MSD Mesoscale® analysis of TFA-soluble (TBS-insoluble) Aβ1:40 and Aβ1:42 levels in combined cortical and hippocampal tissue from vehicle and 1 wk ABX gvg-treated APP_SWE_/PS1_ΔE9_ mice using anti-Aβ mAb, 4G8 (*n* = 14). (**E**) MSD Mesoscale® analysis of TBS-soluble Aβ1:40 and Aβ1:42 levels in combined cortical and hippocampal tissue from vehicle and 1 wk ABX gvg-treated APP_SWE_/PS1_ΔE9_ mice using anti-Aβ mAb, 4G8 (*n* = 14). Aβ concentrations are expressed relative to total protein concentrations obtained from the total-TBS soluble fraction used in the MSD Mesoscale® assay. (**F**) Immunoblot of full length APP (APP-FL) and BACE expression in RIPA-soluble brain lysates of vehicle and 1 wk ABX gvg-treated APP_SWE_/PS1_ΔE9_ mice using anti-APP mAb, 26D6 and an anti-BACE mAb. Samples were run both individually and as pools to confirm expression. Densitometry of (**G**) APP-FL and (**H**) BACE expression as detected by immunoblotting (*n* = 8). All densitometry is expressed as a ratio of APP-FL:β-actin or BACE:β-actin raw pixel intensities. Immuno-detection of β-actin was used to ascertain loading quantities. Full length blots can be viewed in Supp. Fig. 9. Data are displayed as mean ± SEM. See Supp. Figs 7, 8, 9 and statistical Table 2 for additional information.

We further stratified vehicle and 1 wk ABX gvg-treated APP_SWE_/PS1_ΔE9_ mice based on low or high amyloid burden determined by Aβ stereology using an anti-Aβ mAb 3D6 (see Fig. 3), and analysed serum cytokine/chemokine levels using the aforementioned arrays. A noticeable increase in inflammatory milieu was observed in the vehicle-treated high amyloid group compared to the low amyloid group, but this observation was not apparent in the 1 wk ABX gvg group (Supp. Fig. 6). Densitometric analysis of these arrays revealed numerous inflammatory mediators that were up-regulated in serum of low amyloid 1 wk ABX gvg-treated APP_SWE_/PS1_ΔE9_ mice, compared with vehicle, but this difference was attenuated when the same comparison was made in the high amyloid treatment groups (Supp. Fig. 6B). We interpret these findings to suggest that numerous inflammatory mediators in the serum positively correlate with amyloid load and that ABX treatment during post-natal development appears to alter this inflammatory response.

### 1 wk ABX gvg-treated APP_SWE_/PS1_ΔE9_ mice exhibit reduced Aβ plaque deposition in the brain

To investigate the effect of short-term post-natal ABX treatment on amyloidosis in older APP_SWE_/PS1_ΔE9_ mice, we incubated serial sections from hemi-brains of 6.5-month-old vehicle and 1 wk ABX gvg-treated animals with an Aβ-specific monoclonal antibody (mAb) 3D6 and bound antibodies were detected with Alexa Fluor® 488-conjugated donkey anti-mouse IgG. Sections were imaged and Aβ plaque burden was quantified by stereological analysis (Fig. 3A). These studies revealed a significant 1.79-fold decrease in combined cortical and hippocampal Aβ plaque burden in 1 wk ABX gvg-treated APP_SWE_/PS1_ΔE9_ mice compared with age matched vehicle controls (Vehicle: 1.00 ± 0.14% vs. 1 wk ABX gvg: 0.58 ± 0.09%, *p = 0.017, *n* = 12, Fig. 3B). High power × 60 magnification z-stack images of these Aβ plaques also revealed a significant 2.22-fold decrease in the size of individual deposits in 1 wk ABX gvg-treated APP_SWE_/PS1_ΔE9_ mice compared to vehicle controls (Vehicle: 783.2 ± 47.74 µm^2^ vs. 1 wk ABX gvg: 352.4 ± 46.20 µm^2^, *n* = 12, 4 plaques per mouse, *p = 0.011, Fig. 3C).

To validate these immunohistological findings, we removed the striatum from the opposite hemisphere and prepared TBS-soluble and TBS-insoluble fractions from combined cortical and hippocampal tissue to determine levels of Aβ species by MSD MesoScale® ELISA-based assay. We observed trends, albeit statistically nonsignificant, towards decreased levels of TBS-insoluble Aβ1-40 (Vehicle: 390.7 ± 163.4 pg/mg total protein vs. 1 wk ABX gvg: 81.98 ± 15.81 pg/mg total protein, *n* = 14, Fig. 3D) and Aβ1-42 (Vehicle: 4213 ± 1949 pg/mg total protein vs. 1 wk ABX gvg: 728.9 ± 115 pg/mg total protein, *n* = 14, Fig. 3D) peptides in 1 wk ABX gvg-treated APP_SWE_/PS1_ΔE9_ mice compared to vehicle controls. Calculating the ratio of TBS-insoluble Aβ1-42:TBS-insoluble Aβ1-40 revealed no preferential Aβ species present in the brains of 1 wk ABX gvg-treated APP_SWE_/PS1_ΔE9_ mice compared with vehicle controls (Supp. Fig. 7A). Importantly, we observed a clear correlation between 3D6-positive Aβ plaque burden and TBS-Insoluble Aβ levels in both vehicle and 1 wk ABX gvg-treated APP_SWE_/PS1_ΔE9_ mice, thus validating our assays (r^2^(Aβ1:40): 0.76, r^2^(Aβ1:42): 0.79, Supp. Fig. 7B). Collectively, these immunocytochemical and biochemical findings reveal that ABX treatment exclusively during post-natal development reduces Aβ plaque burden in APP_SWE_/PS1_ΔE9_ mice that are assessed at the age of onset for amyloid deposition.

We also assessed the TBS-soluble fraction of these same tissues using the MSD MesoScale® assay. No alterations in TBS-soluble Aβ1-40 (Vehicle: 199.9 ± 36.09 pg/mg total protein vs. 1 wk ABX gvg: 260.7 ± 46.96 pg/mg total protein, *n* = 14, Fig. 3E) or Aβ1-42 levels (Vehicle: 536.3 ± 115.2 pg/mg total protein vs. 1 wk ABX gvg: 604.6 ± 126.8 pg/mg total protein, *n* = 14, Fig. 3E) were detected in the brains of 1 wk ABX gvg-treated APP_SWE_/PS1_ΔE9_ mice compared with vehicle controls. Calculating the ratio of TBS-soluble Aβ1-42:TBS-soluble Aβ1-40 revealed no preferential Aβ species present in 1 wk ABX gvg-treated APP_SWE_/PS1_ΔE9_ mice compared to vehicle controls (Supp. Fig. 7C). As the absolute levels of Aβ1:38 were significantly lower than the levels of Aβ1-42 and Aβ1-40 peptides in the brains of the cohorts being assessed, the variability between samples precluded making any conclusions from this dataset (Supp. Fig. 7D).

In view of our demonstration that 1 wk ABX gvg-treated APP_SWE_/PS1_ΔE9_ mice showed diminished amyloid burden compared with vehicle-treated animals, we considered the possibility that our findings might be the result of decreased transgene or APP-cleavage enzyme expression in the brains of the 1 wk ABX gvg-treated APP_SWE_/PS1_ΔE9_ mice. To clarify this issue, we performed Western blot analysis with the APP-specific mAb 26D6 antibody that detects an epitope within the amino-terminal portion of Aβ, and a BACE-specific, mAb D10E5 antibody that detects an epitope within the carboxyl-terminal region of the polypeptide (Fig. 3F), and performed densitometric quantification of the blots (Fig. 3G,H). These studies revealed no differences in the levels of full length APP or BACE expression in RIPA-soluble brain lysates between vehicle and 1 wk ABX gvg-treated APP_SWE_/PS1_ΔE9_ mice (*n* = 8, Fig. 3G,H). Additionally, we analysed the levels of immature and mature APP and α- and β-secretase-generated C-terminal fragments (CTFs) and the soluble APP ectodomain (sAPPβ_SWE_) generated by β-secretase (Supp. Fig. 8). We observed no differences in levels of full-length APP or it’s metabolites between vehicle and 1 wk ABX gvg-treated APP_SWE_/PS1_ΔE9_ mice. These findings imply that reduction of amyloid plaque burden observed in 1 wk ABX gvg-treated APP_SWE_/PS1_ΔE9_ mice is not the result of alterations in APP proteolysis or β-secretase levels.

### Plaque-localised glial reactivity is reduced in 1 wk ABX gvg-treated APP_SWE_/PS1_ΔE9_ mice

**Figure 4:**
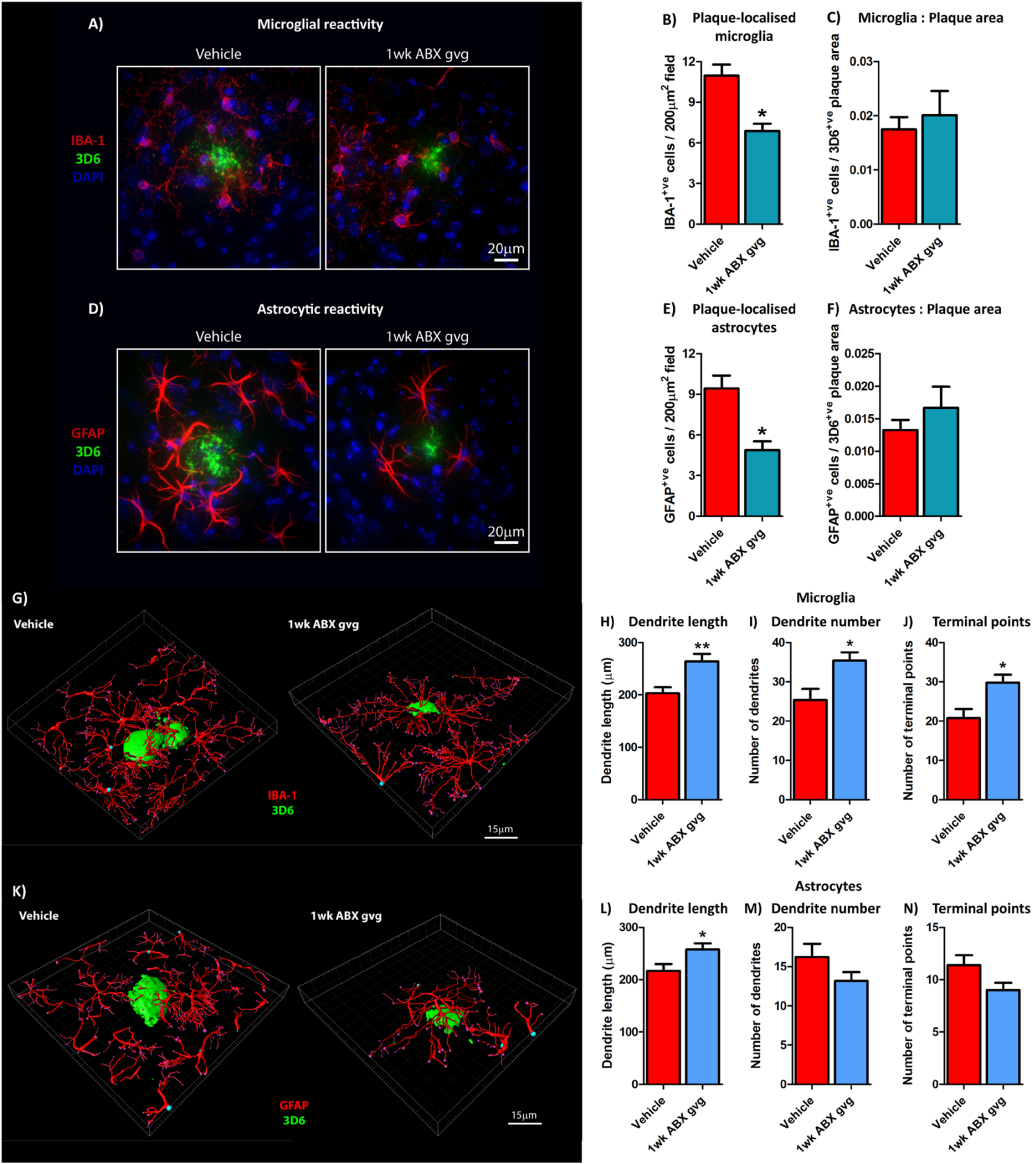
Plaque-localised glial reactivity is reduced in 1 wk ABX gvg-treated APP_SWE_/PS1_ΔE9_ mice. (**A**) Representative ×60 magnification z-stack maximum projection images of IBA-1^+ve^ Aβ plaque-localised microglia, co-stained with DAPI, in vehicle and 1 wk ABX gvg-treated 6.5-month-old APP_SWE_/PS1_ΔE9_ mice. (**B**) Quantification of plaque-localised IBA-1^+ve^ microglial number in vehicle and 1 wk ABX gvg-treated APP_SWE_/PS1_ΔE9_ mice (*n* = 12, *p < 0.05, unpaired two-tailed Student’s *t*-test). (**C**) Plaque-localised IBA^+ve^ microglial number expressed relative to 3D6^+ve^ Aβ plaque area in vehicle and 1 wk ABX gvg-treated APP_SWE_/PS1_ΔE9_ mice (*n* = 12). (**D**) Representative ×60 magnification z-stack maximum projection images of GFAP^+ve^ Aβ plaque-localised astrocytes, co-stained with DAPI, in vehicle and 1 wk ABX gvg-treated APP_SWE_/PS1_ΔE9_ mice. (**E**) Quantification of plaque-localised GFAP^+ve^ astrocyte number in vehicle and 1 wk ABX gvg-treated APP_SWE_/PS1_ΔE9_ mice (*n* = 12, *p < 0.05, unpaired two-tailed Student’s *t*-test). (**F**) Plaque-localised GFAP^+ve^ astrocyte number expressed relative to 3D6^+ve^ Aβ plaque area in vehicle and 1 wk ABX gvg-treated APP_SWE_/PS1_ΔE9_ mice (*n* = 12). (**G**) 3D-IMARIS-based reconstructions of IBA-1^+ve^ plaque-localised microglia and quantification of (**H**) dendrite length, (**I**) dendrite number, and (**J**) terminal points in a subset of vehicle and 1 wk ABX gvg-treated APP_SWE_/PS1_ΔE9_ mice used for cell counts above (*n* = 5, **p < 0.01, *p < 0.05, unpaired two-tailed Student’s *t*-test). (**K**) 3D-IMARIS-based reconstructions of GFAP^+ve^ plaque-localised astrocytes and quantification of (**H**) dendrite length, (**I**) dendrite number, and (**J**) terminal points in these same mice (*n* = 5, *p < 0.05, unpaired two-tailed Student’s *t*-test). Data are displayed as mean ± SEM. See statistical Table 2 for additional information.

To assess potential alterations in the neuro-inflammatory state and reactive gliosis, we analysed Aβ plaque-localised microglial populations by immunohistochemistry using an antibody to ionised calcium binding adaptor molecule 1 (IBA-1), a microglia/macrophage-specific calcium-binding protein^47, 48^ (Fig. 4A). Analysis of high power ×60 magnification z-stack images revealed a significant 1.60-fold decrease in plaque-localised IBA-1-positive microglial cells in 1 wk ABX gvg-treated APP_SWE_/PS1_ΔE9_ mice compared with vehicle controls (Vehicle: 11.73 ± 0.79cells/200 µm^2^ field vs. 1 wk ABX gvg: 6.88 ± 0.54cells/200 µm^2^ field, *n* = 12, *p = 0.011, Fig. 4B). However, this difference was not apparent when the number of IBA-1-positive cells was expressed relative to the area occupied by 3D6-positive plaques (*n* = 8, Fig. 4C).

Recent evidence now suggests that microglial-mediated neuro-inflammatory activity can drive astrocytic reactivity to induce multi-cellular immune responses in the CNS^49^. Hence, we performed immunocytochemistry with an antibody specific for glial fibrillary acidic protein (GFAP) that is highly enriched in astrocytes^50^ (Fig. 4D). Analysis of high power ×60 magnification z-stack images revealed a significant 1.94-fold decrease in plaque-localised GFAP-positive astrocytes in 1 wk ABX gvg-treated APP_SWE_/PS1_ΔE9_ mice compared with vehicle controls (Vehicle: 9.43 ± 0.94 cells/200 µm^2^ field vs. 1 wk ABX gvg: 4.87 ± 0.66cells/200 µm^2^ field, *n* = 12, *p = 0.011, Fig. 4E). Similar to the microglial analysis, this difference was not apparent when the number of GFAP-positive cells was expressed relative to Aβ plaque area (*n* = 12, Fig. 4F). These findings confirm that similar to microglia, astrocyte reactivity surrounding Aβ deposits is reduced in brains of 1 wk ABX gvg-treated APP_SWE_/PS1_ΔE9_ mice.

As cellular morphology is an important determinant of microglial and astrocytic activity, we analysed the structure of plaque-localised microglia and astrocytes in vehicle and 1 wk ABX gvg-treated APP_SWE_/PS1_ΔE9_ mice. In a subset of 1 wk ABX gvg-treated APP_SWE_/PS1_ΔE9_ mice, IMARIS-based reconstructions identified significantly altered microglial morphology (Fig. 4G) when dendrite length (Vehicle: 203.2 ± 11.53 µm vs. 1 wk ABX gvg: 264.4 ± 14.04 µm, *n* = 5, **p = 0.0098, Fig. 4H), dendrite number (Vehicle: 25.4 ± 2.82 vs. 1 wk ABX gvg: 35.4 ± 2.11, *n* = 5, *p = 0.0219, Fig. 4I) and total terminal points (Vehicle: 20.80 ± 2.25 vs. 1 wk ABX gvg: 29.80 ± 2.01, *n* = 5, *p = 0.0174, Fig. 4J) were quantified. It is important to note that this observed change in microglial morphology was not observed in 50% of the entire cohort analysed (data not shown), suggesting heterogeneity between mice or response to 1 wk ABX gvg treatment. On the other hand, we observed more uniform alterations in astrocyte morphology using this same technique (Fig. 4K). Quantification of dendrite length revealed significant alterations between 1 wk ABX gvg-treated and vehicle-treated APP_SWE_/PS1_ΔE9_ mice (Vehicle: 216.6 ± 13.26 µm vs. 1 wk ABX gvg: 257.5 ± 11.55 µm, *n* = 5, *p = 0.0485, Fig. 4L), but no differences were detected when dendrite number (Fig. 4M) and total terminal points (Fig. 4N) were analysed. These findings suggest that microglial and astrocytic morphology is altered in response to pre-weaning ABX treatment that may impact on cellular activity surrounding amyloid deposits.

## Discussion

It is becoming increasingly evident that the commensal microbiome plays a complex role in regulating host innate immunity and brain physiology in health and disease^30, 31^. In the context of AD, where neuro-inflammatory responses play a significant role in the progression of disease, *Escherichia* and *Shigella* belonging to pro-inflammatory gut bacterial taxa, have been positively correlated with inflammatory burden in AD patients^34^ and Aβ-overexpressing transgenic mouse models of the disease harbor a distinct gut microbial profile in comparison to non-transgenic littermates^36^. Additionally, GF Thy1-APP_SWE_/PS1_L166P_ mice display attenuated amyloidosis and dampened neuro-inflammatory responses^36^. We previously reported that perturbations in the microbial diversity of the APP_SWE_/PS1_ΔE9_ amyloidosis murine model of AD induced by life-long ABX treatment, is associated with an altered peripherally circulating inflammatory mediator milieu that parallels an attenuation of Aβ deposition and reduced plaque-localised gliosis^35^. Both the aforementioned studies using GF and ABX-treated APP/PS1 mice^35, 36^ maintain microbial absence or selection pressure during development and throughout the life span, however a strong body of evidence suggests the post-natal development period alone presents a critical window by which microbiota-host interactions shape immunity and brain function in later life^41, 43–45^.

The present study was designed to test the hypothesis that significant microbial perturbations during post-natal developmental alone may be sufficient to recapitulate the altered phenotypes observed in APP_SWE_/PS1_ΔE9_ mice that were subjected to life-long ABX selection pressure^35^. We now offer several novel insights. First, we demonstrate that a 1 wk ABX gvg treatment regimen induces significant expansion of *Lachnospiraceae* and contraction of *S24-7* that is maintained throughout life, albeit generalised microbial diversity perturbations are minor compared to our earlier findings in animals treated with a life-long ABX treatment regimen^35^. While the metabolic pathways of *S24-7* commensals are only beginning to be elucidated^51^, *Lachnospiraceae* are robust butyrate-producers^52, 53^ and this may induce alterations in T-cell differentiation^27, 28^ and microglial function^29^. Second, we observe an upregulation of circulating and brain-residing Foxp3^+^ T-regs with altered sera and CSF cytokine/chemokine composition. Thirdly, we demonstrate a significant reduction in amyloid plaque deposition in brains of 1 wk ABX gvg-treated APP_SWE_/PS1_ΔE9_ mice. Finally, 1 wk ABX gvg-treated APP_SWE_/PS1_ΔE9_ mice show attenuated neuro-inflammatory responses as revealed by reduced Aβ plaque-localised gliosis in these mice.

Our demonstration that 1 wk ABX gvg-treatment of APP_SWE_/PS1_ΔE9_ mice leads to promotion of blood-circulating and brain-residing Foxp3^+^ T-regs is notable. Lymphoid cell populations are closely regulated by the commensal microbiota composition^24^ and can circulate to the brain where they exhibit type-1/2 IFN-dependent inflammatory activity^54^. Curiously a type-1 IFN signature has been implicated in AD progression^55, 56^ and T-cell trafficking is considered a primary mechanism to facilitate peripheral and central immune-communication in AD^13^. Indeed, re-population of Foxp3^+^ T-regs post-transient deletion is sufficient to re-balance or re-program neuro-inflammatory responses and reduces amyloid deposition in 5xFAD mice^20^. IL-2-induced proliferation of Foxp3^+^ T-regs alleviates cognitive deficits in Thy1-APP_SWE_/PS1_L166P_ mice^18^ and adoptive transfer of these cells reduces cortical and hippocampal Aβ plaque deposition in 3xTg FAD mice^19^. Our observation of elevated Foxp3^+^ T-regs correlating with attenuated Aβ plaque and plaque-localised gliosis in 1 wk ABX gvg-treated APP_SWE_/PS1_ΔE9_ mice supports the notion that microbial perturbations can regulate peripheral immune mechanisms and potentially impact Aβ clearance mechanisms. We propose two potential mechanisms for the elevated Foxp3^+^ T-reg populations present within 1 wk ABX gvg-treated APP_SWE_/PS1_ΔE9_ mice. First, the observed expansion of gut-residing *Lachnospiraceae* may result in increased butyrate production^52, 53^ that diffuses into the host circulatory system and is known to induce T-reg differentiation^27, 28^. Second, the observed elevation in IL-2 expression in the sera of 1 wk ABX gvg-treated APP_SWE_/PS1_ΔE9_ mice has the capacity to drive direct amplification of Foxp3^+^ T-regs that appears beneficial in APP_SWE_/PS1_L166P_ mice^18^. Finally, we observe reduced levels of the pro-inflammatory cytokine, IL-6 that has been implicated in AD^57–59^, in serum of 1 wk ABX gvg-treated APP_SWE_/PS1_ΔE9_ mice. Thus the IL-2:IL-6 ratio likely favours the differentiation of T-regs as opposed to pro-inflammatory Th17 cells. The evident Foxp3^+^ T-reg expansion is likely the product of these mechanisms alongside additional factors and supports the notion that commensal microbes and, importantly, their immunologically-active metabolites can influence host immunity.

In conjunction with alterations in T cell populations, we also observed differentially regulated cytokine/chemokine profiles in both the sera and CSF of 1 wk ABX gvg-treated APP_SWE_/PS1_ΔE9_ mice. SCF is a potent hematopoietic growth factor stimulating neurogenesis^60^ and is known to protect against ischemic stroke *in vivo* ^61, 62^. Interestingly, fast-progressing AD patients display diminished SCF serum concentrations^63^ and combined sub-cutaneous SCF/G-CSF treatment in APP_SWE_/PS1_L166P_ mice attenuates cortical and hippocampal Aβ deposition^64^. While our observation of elevated SCF serum concentration in 1 wk ABX gvg-treated APP_SWE_/PS1_ΔE9_ mice displaying attenuated plaque deposition would support these studies, these mice also exhibited dramatically down-regulated SCF expression in the CSF. Both the serum and CSF compartments maintain access to different immune barriers of the brain, namely the blood-brain barrier and blood-cerebrospinal fluid-brain barrier, respectively and may impart differential inflammatory actions on the brain. Considering SCF is one inflammatory entity that is differentially expressed in the serum and CSF of 1 wk ABX gvg-treated APP_SWE_/PS1_ΔE9_ mice, further compartmental-based investigations are required to elucidate the impact of these altered inflammatory profiles on neuro-inflammation and amyloidosis.

We were particularly intrigued by the observation that serum levels of CCL11 are elevated in the serum of 1 wk ABX gvg-treated animals, a finding consistent with our earlier studies in APP_SWE_/PS1_ΔE9_ mice subjected to life-long ABX selection^35^. CCL11 has been linked to age-associated deficits in hippocampal neurogenesis^65^, and the chemokine gene cluster containing CCL11 has recently been implicated as a risk factor for late-onset AD^66^. CCL11 is known to cross the blood brain barrier^67^ and we speculate, that alongside enhanced Foxp3^+^ T-reg activity and interleukin expression, would lead to microglial activation and subsequent phagocytosis of oligomeric or deposited forms of Aβ in relatively young APP_SWE_/PS1_ΔE9_ mice being analysed here. In this manner, CCL11 is a persistent chemokine expressed during macrophage-mediated phagocytosis of myelin^68^ suggesting this mediator is permissive to cellular clearance mechanisms that are shared by microglia. We maintain the notion that temporal elevations of cytokine and chemokine expression initiates a beneficial early-stage neuro-inflammatory response that facilitates Aβ peptide clearance, a phenotype that might convert to a dysregulated pro-inflammatory state that could exacerbate AD progression. In this manner, we are currently conducting RNA-sequencing studies to ascertain the inflammatory transcriptomic profile of microglia isolated from ABX-treated APP_SWE_/PS1_ΔE9_ mice prior to and during age-of-onset for Aβ plaque deposition. GF mice display remarkably altered microglial transcriptomic profiles controlling cellular surveillance and response to bacterial or viral challenge^29^, and hence we predict that our future transcriptomic studies will provide invaluable insight into microglial activity in the context of amyloidosis in microbially perturbed hosts.

We fully understand the correlative nature of this study and do not clarify the precise mechanism(s) responsible. Nevertheless, our findings indicate that ABX-induced perturbations in microbial diversity during post-natal development stages alone is associated with altered host immune parameters and confers attenuated Aβ amyloidosis in a manner similar to that observed in APP_SWE_/PS1_ΔE9_ mice subjected to life-long ABX selection pressure^35^. These findings, in conjunction with aforementioned studies using GF mice^33, 36^, suggest that developmental periods provide a crucial time-frame by which the commensal microbiome shapes host immunity and maintains important implications for neurodegenerative diseases. It will be critical to further investigate these observations to further understand the precise mechanism(s) involved. Evidence implies that specific microbially-derived metabolites can directly interfere with Aβ oligomerisation^69^ and identifying Aβ-interacting or immunologically active metabolites in these mice using metabolomics profiling techniques will be of great interest. The answers to these challenging issues that we will endeavour to address in future studies, we hope to offer new insights into the regulation of Aβ plaque deposition by microbially-regulated immune responses. Ultimately, these findings may lead to targeted microbiome-based interventions for the treatment of early stage Alzheimer’s disease.

## Methods

### Animals and antibiotic treatment regime

All murine experimental procedures were approved by the Institutional Animal Care and Use Committee (IACUC) at the University of Chicago and performed in accordance with approved Animal Care and Use Protocols (ACUPs).

Specific pathogen free male APP_SWE_/PS1_ΔE9_ transgenic mice on a hybrid C57BL/6-C3-hej genetic background (B6C3-Tg(APPswe,PSEN1dE9)85Dbo/Mmjax, JAX ID: 004462, www.jax.org/strain/004462) were housed in sterile micro-isolator cages and fed *ad-libitum* on standard chow. A portion of cage bedding from sentinel-housed mice was placed within each micro-isolator cage housing new born litters to ensure similar microbial colonisation prior to treatment stratification. All newly-introduced materials into micro-isolator cages ere sterilised by autoclaving. Mice were then gavaged once-daily with combinatorial antibiotics (ABX, gentamicin (1 mg/ml), vancomycin (0.5 mg/ml), metronidazole (2 mg/ml), neomycin (0.5 mg/ml), ampicillin (1 mg/ml), kanamycin (3 mg/ml), colistin (6000U/ml), and cefaperazone (1 mg/ml) diluted in autoclaved water, Sigma) from post-natal day 14 until day 21 when pups were weaned onto standard drinking water (1 wk ABX gvg)^35, 70^. Mice assigned to vehicle control groups were subjected to the same treatment protocol with autoclaved water only. Mice were sacrificed at 6.5 months of age for subsequent experimentation (Fig. 1A, Supp. Fig. 1A).

### Monitoring 1 wk ABX gvg treatment efficacy

Fresh faecal pellets were obtained from animals and homogenised in 1 ml phosphate-buffered saline (PBS) via repetitive pipetting under aseptic conditions. Homogenates were then filtered through sterile 70 µm pore-sized cell strainers (BD Biosciences) and spread on Brucella blood agar plates (Invitrogen), sealed with laboratory parafilm and incubated overnight at 37 °C under anaerobic conditions. Faecal homogenates were also grown in 5 ml Luria broth (LB, Invitrogen) medium overnight in aerobic conditions at 37 °C with shaking (225 rpm) and optical density (O.D.) of cultures was measured at 600 nm using an ELx808 multi-channel absorbance reader (BioTek) to track bacterial growth rates. Efficacy of the 1 wk ABX gvg treatment was confirmed by monitoring anaerobic bacterial growth on Brucella blood agar plates and aerobic growth in LB media throughout the treatment course (Supp. Fig. 1B,C).

### Cardiac perfusion of mice and tissue harvesting

Mice were deeply anaesthetised using inhaled isoflurane, USP (Piramal Healthcare) and were cardiac perfused with ice-cold heparinised PBS (1U/ml, Sigma).

Blood was obtained directly from the lacerated right atrial outlet using ethylenediaminetetraacetic acid (EDTA)-coated syringes and micro-centrifuge tubes to prevent clotting. Blood was then centrifuged (10,000xg, 10 mins, 4 °C) and the serum-containing supernatant was snap frozen in liquid nitrogen and stored at −80 °C until required.

Brains were then excised and hemispheres were separated for use in immunohistochemistry or for protein biochemical analysis. Isolation of the cortex for protein biochemistry was performed using a modified dissection technique^71^. Hemispheres were placed on an ice cold glass dissection plate and orientated in a sagittal plane. The cerebellum, striatum, thalamus, midbrain and brain stem were then removed using sterilised blunt spatulas, exposing the hippocampal complex and interior wall of the cortex. Combined cortical and hippocampal tissue was snap frozen in liquid nitrogen and stored at −80 °C until required. For immunohistochemistry, the opposing hemispheres were post-fixed in 4% w/v paraformaldehyde, 0.1% v/v glutaraldehyde for 72 hrs, submerged in 30% w/v sucrose for cryoprotective purposes and stored at 4 °C until required.

The GI tract was dissected and removed from surrounding adiposity in ice-cold sterile PBS. Sections of the ileum, proximal colon and distal colon were placed in histology cassettes (Fisher Scientific) and fixed in Carnoy’s solution (60% v/v methanol, 30% v/v chloroform, 10% glacial acetic acid, 3 hrs, room temperature). Cassettes were then stored in 70% v/v ethanol at room temperature until required. Cecal contents were also obtained and were snap frozen in liquid nitrogen followed by storage at −80 °C until required.

### Isolation of cerebrospinal fluid from cisterna magna

An individual cohort of vehicle and 1 wk ABX gvg-treated APP_SWE_/PS1_ΔE9_ mice was used for CSF extraction as previously described^72^. Briefly, mice were deeply anaesthetised using ketamine (150 mg/kg, Vedco)/Xylazine (10 mg/kg, Sigma Aldrich) administered in an intra-peritoneal (IP) manner. The neck of the anesthetised mouse was then shaved and placed on a stereotaxic frame whereby the head of the mouse was dropped to a 45 °C decline and secured. The subcutaneous tissue and muscle layers were then separated by blunt dissection until the cisterna magna was clearly visible in close proximity to the *arteria dorsalis spinalis*. Using a sterile cotton swab submerged in PBS, the dissection area was cleaned and cauterised to prevent contamination of CSF sampling by residual blood or extracellular fluid. The cisterna magna was then carefully pierced with a 30 gauge needle and CSF was collected using pre-chilled pipette tips and micro-centrifuge tubes. Each CSF sample was then examined under a dissecting microscope and briefly centrifuged (17,000 × g, 10secs, 4 °C) to confirm absence of red blood cell contamination. CSF was then snap frozen in liquid nitrogen and stored at −80 °C until required.

### Cecal and Fecal DNA extraction

Extraction of bacterial DNA from caecal and faecal contents was performed as previously described^73^ and conforms to standardised earth microbiome project protocols (www.earthmicrobiome.org/emp-standard-protocols/). Briefly, tissues were dissolved in extraction buffer (50 mg tissue/ml buffer, 50 mM Tris (pH 7.4), 100 mM EDTA (pH 8.0), 400 mM NaCl, 0.5% w/v SDS) containing proteinase K (0.4 mg/ml). After addition of 0.1-mm diameter glass beads (500ul/ml buffer, BioSpec Products), microbial cells were lysed using a Mini-Beadbeater-8K Cell Disrupter (BioSpec Products) and overnight water bath incubation (55 °C). Total DNA was then extracted using Phenol:Chloroform:IAA (25:24:1 v/v, pH 8.0, Ambion) according to manufacturer’s protocols. DNA yield was quantified and quality ascertained by a combination of Nanodrop Lite (Thermo Fisher) and Qubit® fluorometer (Invitrogen) assessment.

### 16 s rRNA gene quantitative polymerase chain reaction (Q-PCR)

16 s rRNA gene copy number was quantified from DNA isolated from faecal and caecal contents using Q-PCR. 2–5 ng/ul DNA was added to iQ-SYBR green PCR supermix (BioRad) with 518 F (5′-TCC-TAC-GGG-AGG-CAG-CAG-T-3′) and 338 R (5′-GGA-CTA-CCA-GGG-TAT-CTA-ATC-CTG-TT-3′) primers (2.5 µM) and Q-PCR was performed on a Lightcycler® 96 system (Roche) under the parameters listed in Table 1.

**Table 1:** Thermal cycler conditions for 16 s rRNA SYBR green Q-PCR.

Step	Temperature	Time	Repeats
1	95 °C	5 min	1
2	95 °C	10 sec	35
3	64 °C	45 sec	
4	72 °C	45 sec	
5	40 °C	30 sec	1

16 s rRNA gene copy number was determined by reference of Cp values to a standard curve of the pCR4-TOPO plasmid inclusive of the 16 s rRNA gene amplicon. Copy number was then expressed relative to the DNA concentration added per reaction as determined by earlier Qubit® fluorometer (Invitrogen) assessment. All reactions were conducted in triplicate in a 96-well plate format (white-walled, Roche) with appropriate negative controls.

### 16 s rRNA gene PCR and terminal restriction fragment length polymorphism analysis

T-RFLP analysis of the 16 s rRNA gene product from cecal and fecal bacterial DNA was performed as previously described^73^. Briefly, the 16 s rRNA gene was amplified using primers (0.25 µM) 8 F (5′-AGA-GTT-TGA-TCC-TGG-CTC-AGT-3′), labelled with 6′-FAM at the 5′ end, and 1492 R (5′-GGT-TAC-CTT-GTT-ACG-ACT-T-3′) in conjunction with the Takara® Ex-Taq polymerase system (Takara) under the parameters listed in Table 2.

**Table 2:** Thermal cycler conditions for 16 s rRNA gene amplification.

Step	Temperature	Time	Repeats
1	94 °C	10 min	1
2	94 °C	30 sec	30
3	55 °C	30 sec	
4	72 °C	90 sec	
5	72 °C	7 min	1

PCR amplification of samples was confirmed by DNA electrophoresis using 1% w/v agarose (Sigma) gels. DNA was then precipitated (ethanol/sodium acetate) and digested with the MspI restriction enzyme in NEBuffer 4 (1333U/ml, New England Biolabs) at 37 °C for 4 hours. Reactions were terminated at 65 °C (10 mins) and dialysed on VSWP filters (Millipore) before mixture with GeneScan-500 Size Standard (Applied Biosystems) and subsequent sequencing on a 96-capillary 3730 DNA analyzer (Applied Biosystems/Hitachi, University of Chicago comprehensive cancer center).

Individual fragment lengths were then determined relative to the GeneScan-500 Size Standard and calculated using Gene Mapper® v4.1 software (Applied Biosystems). The additive main effects and multiplicative interaction model (AMMI, doubly-centred principal component analysis (PCA)) was then applied to the dataset using T-REX freeware (trex.biohpc.org) to generate PCA plots.

### 16 s rRNA gene Illumina® MiSeq sequencing

Sequencing of the 16 s rRNA gene from caecal and faecal contents was performed as previously described^74^. The V4-V5 amplicon region of the 16 s rRNA gene was amplified using standard PCR methods (www.earthmicrobiome.org/emp-standard-protocols) from total DNA. Illumina® MiSeq gene sequencing was then performed within the Institute for Genomics and Systems Biology’s Next Generation Sequencing Core at Argonne National Laboratory. Sequences were trimmed and classified using QIIME version 1.7^75^. Operational taxonomic units (OTUs) were picked at 97% sequence identity using open-reference OTU picking against the Greengenes database version 12_10^76^. These quality-controlled sequences were then aligned using PyNAST^77^, taxonomy was assigned using the RDP classifier^78^ and a phylogenetic tree was constructed using FastTree version 2.0^79^. Shannon indices, indicative of α-diversity were then calculated from rarefaction plots based on the number of quality sequences obtained from each sample. Un-weighted and weighted UniFrac distances were then computed to produce β-diversity dissimilarity matrices^80^ and PCA plots were generated.

### Analysis of immune cell compartments by flow cytometry

An individual cohort of vehicle and 1 wk ABX gvg-treated APP_SWE_/PS1_ΔE9_ mice was used for flow cytometry analysis. Blood was collected via cheek bleed in EDTA-coated tubes and red blood cells were lysed by incubation with ice-cold ammonium chloride (10 mins, room temperature incubation, Stem cell technologies). Mice were then sacrificed by cervical dislocation and mesenteric lymph nodes (MLNs) were collected and mashed through a 100 µm cell strainer (BD Biosciences) to obtain a single cell suspension. Brains were dissected from the skull, rinsed in ice-cold PBS and mashed using a glass dunce homogeniser. Brain homogenates were digested in Roswell Park Memorial Institute medium (RPMI, Corning) containing Collagenase D (2.5 mg/ml w/v, Roche) and DNAse-I (1 mg/ml w/v, Roche) for 40 minutes at 37 °C. Mononuclear cells of the brain were isolated by passing the tissue through a 70 µm cell strainer, followed by a 70%/30% sterile Percoll™ (GE Healthcare) gradient centrifugation (20 mins, room temperature, 300xg, no breaking) as previously described^81^. Mononuclear cells were removed from the interphase and washed two times with ice-cold PBS containing 2% v/v FBS.

Cell preparations were analysed for intra-nuclear levels of Foxp3, T-bet, RorγT, GATA-3 or cytokine expression of IFNγ, IL-17, IL-4 and TNFα by flow cytometry. For detection of cytokines, cells were stimulated in RPMI containing FBS (10% v/v), phorbol 12-myristate 13-acetate (PMA, 50 ng/ml w/v, Sigma), ionomycin (500 ng/ml w/v, Sigma), Golgi Stop (1.3 µl/ml v/v, BD Biosciences) and Golgi Plug (1 µl/ml v/v, BD Biosciences) for 3 hours (37 °C, 5% CO_2_) prior to staining. Cells were then incubated in Fc Block^TM^ for 5 mins and Aqua LIVE/DEAD® for 10 mins prior to a 20 min period of surface antibody incubation. Cell samples were then permeabilised with the Foxp3 fixation/permeabilisation kit (45 mins, 4 °C, eBioscience) prior to transcription factor and cytokine staining (30 mins, 4 °C). Flow cytometry was conducted using a 9-color BD FACS Canto instrument (BD Biosciences) with subsequent data analysis and fluorescence gating (Supp. Fig. 2) performed using FlowJo software (Treestar). A list of antibodies and reagents used for flow cytometry is provided in Table 3.

**Table 3:** Antibodies used in flow cytometry analysis.

Antigen/reagent	Conjugated Fluorophore	Isotype (clone)	Dilution factor	Catalogue # (distributor)
T-bet	eFluor® 660	Mouse IgG1 (4B10)	1:100	50-5825-82 (eBioscience)
Foxp3	FITC	Rat IgG2a (FJK-16s)	1:100	11-5773-82 (eBioscience)
RorγT	PE	Rat IgG2a (AFKJS-9)	1:100	12-6988-82 (eBioscience)
GATA-3	PE-cy™7	Rat IgG2b (TWAJ)	1:100	25-9966-42 (eBioscience)
IFNγ	APC	Rat IgG1 (XMG1.2)	1:200	17-7311-81 (eBioscience)
IL-17A	PE	Rat IgG2a (eBio17B7)	1:200	12-7177-81 (eBioscience)
IL-4	AlexaFluor® 488	Rat IgG1 (11B11)	1:200	53-7041-82 (eBioscience)
TNFα	PE-cy™7	Rat IgG1 (MP6-XT22)	1:200	25-7321-82 (eBioscience)
CD4	Per-CP™5.5	Rat IgG2a (RM4-5)	1:200	550954 (BD Biosciences)
TCRβ	APC-cy™7	Armenian Hamster IgG2 (H57-597)	1:200	560656 (BD Biosciences)
CD8a	BV605	Rat IgG2a (53-6.7)	1:200	563152 (BD Biosciences)
CD16/32 (Fc Block™)	N/A	Rat IgG2b (2.4G2)	1 µg/million cells	553141 (BD Biosciences)
CD45	Pacific blue™	Rat IgG2b (30-F11)	1:400	103126 (Biolegend)
Aqua LIVE/DEAD®	Dye (367/526 nm)	N/A	1 µl/million cells	L34957 (Life Technologies)
Foxp3 permeabilisation kit	N/A	N/A	N/A	00-5523-00 (eBioscience)

### Immunohistochemistry

Post-sucrose immersion for cryoprotective purposes, hemispheres were placed in OCT freezing medium and mounted on a Leica SM2000R freezing microtome stage (Leica Biosystems). Hemispheres were then serially sectioned at 40 µm thickness in a coronal plane through the hippocampal complex from −1.5 to −3.0 bregma coordinates (Paxinos G, Mouse brain atlas) and sections were stored in cryoprotective solution (25% v/v glycerin and 30% v/v ethylene glycol in PBS, Fischer Scientific) at −20 °C until required. Every 6^th^ section (spaced 240 µm apart) was used for staining and subsequent quantification.

Sections were heated to 95 °C in SSC buffer (0.15 M Sodium Chloride, 0.15 M Sodium Citrate, pH7.0) for 20 min to aid in antigen presentation and then blocked in 5% v/v donkey serum in Tris-buffered saline with Triton X-100 (TBS-T, 0.25% v/v Triton X-100, Sigma) for 2 hrs at room temperature. Sections were then incubated with unconjugated primary antibodies for 24 hrs at 4 °C. After washing with TBS-T, sections were then incubated with fluorophore-conjugated secondary antibodies for 3 hrs at room temperature whilst protected from light. After sequential washing with TBS-T, TBS and DEPC-treated H_2_O, sections were mounted on Superfrost® plus slides (Fisher Scientific) using VectaShield® mounting medium containing DAPI (Vecta Laboratories). The primary antibodies used were a mouse monoclonal anti-Aβ (3D6, 190 pg/ml final concentration, in-house purified), a rabbit polyclonal anti-ionized calcium-binding adapter molecule 1 (IBA-1, 1:500, 019-19741, Wako) and a rabbit monoclonal anti-glial fibrillary acidic protein (GFAP, 1:2000, clone D1F4Q, 12389 S, Cell Signaling). The fluorophore-conjugated secondary antibodies used were a donkey anti-mouse IgG Alexa Fluor® 488 conjugate (1:1000, A21202, Life Technologies) and a donkey anti-rabbit IgG Alexa Fluor® 594 (1:1000, A21207, Life Technologies). Whole sections were then imaged at ×20 magnification using the Panoramic SCAN BF plus FL Optimum slide scanner (PerkinElmer/3DHistech) or individual plaques at ×60 magnification using the Olympus IX2-series spinning DSU confocal inverted microscope (Olympus).

Carnoy’s-fixed GI tract sections were embedded in paraffin wax, cross-sectioned at 5 µm thickness using a sliding microtome (Leica Biosystems) and direct-mounted onto Superfrost™ Plus slides (Fisher Scientific) within the human tissue resource center (HTRC) at the University of Chicago. Sections were then de-paraffinised, re-hydrated and stained with haematoxylin and eosin. Whole cross-sections were then imaged at ×20 magnification using the Panoramic SCAN BF plus FL Optimum slide scanner (PerkinElmer/3DHistech).

### Amyloid plaque quantification and cell counting

To quantify plaque burden, brain sections prepared as above, were analysed as 8-Bit images using ImageJ software (NIH). For each section intensity thresholds were set to eliminate background fluorescence and plaque staining was analysed by particle quantification. This value was then expressed relative to the calculated area of the combined cortical and hippocampal region of each individual section. This value was then averaged from a minimum of 4 sections per mouse to calculate a plaque burden percentage for each mouse throughout the study. Individual plaque size was calculated using a similar method but also directly from ×60 z-stack images. Plaque-localised microglial and astrocyte cell numbers were counted using a combination of ImageJ and Stereo Investigator (MBF Bioscience) software packages. IBA-1^+ve^ and GFAP^+ve^ positive somas co-localised with DAPI and within the 200 µm vicinity of 3D6^+ve^ Aβ plaque fluorescence were identified as plaque-localised microglia and astrocytes respectively.

### 3D IMARIS-based cell reconstructions

Sections co-labelled with anti-Aβ, anti-IBA-1, and anti-GFAP antibodies and nuclear-stained with DAPI were prepared as above. Z-stack images of individual plaques from 40 µm thick sections were obtained under ×60 magnification and water immersion with 0.75 µm step increments in the *z* plane. These z-stack images were then recorded and analysed using IMARIS software (Bitplane). The surface tool was used to establish the realm of the Aβ plaque and then the filament tool was used to map microglial and astrocytic cell bodies and dendrite-like processes. Quantification of average dendrite length, dendrite number and terminal endpoints was performed with the in-built statistical analysis program. Four Aβ plaque-containing microenvironments were analysed per mouse.

### MSD Meso Scale® Aβ ELISA

For quantification of soluble and insoluble Aβ levels, brain tissues were ground on LN_2_ and were homogenised in TBS containing protease inhibitors (Roche) via sonication and polytron processing. After centrifugation (100,000 × g, 60 min, 4 °C) the supernatant was collected to detect TBS soluble Aβ levels. The remaining TBS-insoluble tissue was further homogenised in 70% v/v formic acid in TBS (TFA, Sigma) via polytron processing, centrifuged (100,000 × g, 60 min, 4 °C), and the resulting supernatant was collected to detect TBS-insoluble Aβ levels. TFA samples were neutralised in 1 M Tris buffer (20x volume) prior to Meso Scale® analysis.

Aβ levels were quantified using Meso Scale® Aβ38/40/42-triplex kits (V-PLEX Aβ Peptide Panel 1 (anti-Aβ mAb, 4G8) Kit, K15199E-1, Meso Scale Diagnostics), as previously described^82–84^. Electro-chemiluminescence signals were captured by the MESO QuickPlex SQ 120 system (Meso Scale Diagnostics). Sample Aβ1:38, Aβ1:40 and Aβ1:42 levels were normalised to the Aβ standard curve and concentrations are expressed relative to sample total protein concentrations as determined by BCA assay (Thermo Fisher). Whilst, Aβ1:40 and Aβ1:42 proteins were robustly detected in all samples assayed, Aβ1:38 was only detected in a subset of vehicle and 1 wk ABX gvg-treated APP_SWE_/PS1_ΔE9_ mouse tissue homogenates (Supp. Fig. 6D).

### Inflammatory cytokine and chemokine array

To analyse the inflammatory mediator profiles from isolated serum and CSF of APP_SWE_/PS1_ΔE9_ mice a RayBio® C-series mouse cytokine antibody array-C3 (AAM-CYT-3-4, RayBiotech) was used according to manufacturer’s instructions. Briefly, membranes containing capture antibodies were blocked in supplied blocking buffer (30 min, room temperature) and samples (Pooled serum diluted 1:2 v/v in blocking buffer or pooled CSF diluted 1:10 v/v in blocking buffer) were incubated overnight at 4 °C. Upon washing with supplied wash buffers, membranes were then incubated with a biotinylated antibody cocktail overnight at 4 °C. After repeated washes, membranes were then further incubated with streptavidin conjugated to horseradish peroxidase (HRP, 2 hrs, room temperature), washed thoroughly again and then exposed to enhanced chemiluminescent (ECL) substrate (2 mins, room temperature). Chemiluminescent images were captured by exposure to X-ray film (MidSci, 30–90 secs) and subsequent photographic development (Kodak).

For densitometry, X-ray film images were scanned and imported into ImageJ (NIH) as 8-Bit images. Background-normalised pixel intensities of regions of interest were calculated in arbitrary units. Inter-membrane intensities were normalised based on the intensity of positive control reactions on each array that maintain a known concentration of printed biotinylated antibody. Expression levels of inflammatory mediators detected in the 1 wk ABX gvg treatment group were then calculated relative to intensities observed from vehicle-treated sample membranes (Supp. Fig. 5).

### Glycine SDS-PAGE, Tricine SDS-PAGE, Western blotting and densitometry

For Western blot analysis, brain tissue was ground in LN_2_ and homogenised in radioimmunoprecipitation assay (RIPA) buffer (50 mM Tris, 150 mM NaCl, 0.1% w/v SDS, 0.5% w/v sodium deoxycholate, 1% v/v Triton X-100) with protease and phosphatase inhibitors (Roche) via sonication. Samples were rotated at 4 °C for 2 hr, centrifuged (13,000 g, 5 min, 4 °C), and protein concentration of the supernatant was determined by BCA assay (Thermo Fisher).

For glycine SDS-PAGE, samples were denatured at 95 °C in reducing buffer (20 mM Tris, 20% v/v glycerol, 4% w/v SDS, 10% β-mecaptoethanol, and bromophenol blue) prior to loading 25 µg of protein onto 12% SDS-PAGE gels (60 mM Tris, 0.1% w/v SDS, 0.1% w/v APS, 0.01% v/v TEMED, 12% v/v Acrylamide/Bis). For tricine SDS-PAGE, samples were denatured as above prior to loading 50 µg of protein onto 10-16.5% SDS-PAGE gels (1 M Tris, 0.1% w/v SDS, 13.33% v/v glycerol, 10-16.5% v/v Acrylamide/Bis, 0.1% w/v APS, 0.01% v/v TEMED). Gel electrophoresis was conducted in SDS-PAGE running buffer (Amresco), for glycine SDS-PAGE, or 0.1 M tris-tricine/0.1% w/v SDS buffer and then a wet transfer in Tris-glycine buffer (Amresco) onto a 0.2 µm nitrocellulose membrane (BioRad) was performed.

Membranes were then blocked in 5% w/v non-fat milk powder in PBS-Tween-20 (0.05% v/v, PBS-T) for 1 hr at room temperature and then incubated with primary antibodies diluted in 2% w/v non-fat milk powder in PBS-T overnight at room temperature (BioRad). Upon washing with PBS-T membranes were then incubated with HRP-conjugated secondary antibodies for 1 hr at room temperature and chemiluminescent signals were produced by incubation with Western Lightning® Plus ECL (PerkinElmer). Chemiluminescent images were captured by exposure to X-ray film (MidSci) and subsequent photographic development (Kodak). Primary antibodies used were a mouse monoclonal anti-APP (26D6, 16 pg/ml final concentration, in-house purified), a rabbit monoclonal anti-BACE (1:1000, 5606, Cell Signalling), a mouse monoclonal anti-APP-CTFs (C1/6.1, 1:500, 802801, Biolegend), a rabbit polyclonal raised against the neo-epitope generated by β-secretase-mediated cleavage of APPswe (192_SWE_, 1:500, S. Sinha of Elan Pharmaceuticals now acquired by Periggo) and a mouse monoclonal anti-β-actin (1:40,000, A5441, Sigma). Secondary antibodies used were a HRP-conjugated goat anti-mouse IgG (1:5000, 31430, Thermo Fisher) and a HRP-conjugated goat anti-rabbit IgG (1:5000, 31460, Thermo Fisher).

For densitometry, X-ray film images were scanned and imported into ImageJ (NIH) as 8-Bit images. Background-normalised pixel intensities of bands of interest were calculated in arbitrary units and expressed relative to that of the β-actin loading control. Data from the 1 wk ABX gvg treatment group was then expressed as fold change relative to the vehicle control group.

### Statistical analysis

GraphPad Prism software (version 6.0, www.graphpad.com/scientific-software/prism/) was used for all un-paired two-tailed Student’s *t*-tests, one-way analysis of variance (ANOVA) with Tukey’s multiple comparisons post hoc tests and Pearson’s multi-variate linear regression analysis. For all statistical tests a two-tailed α value of 0.05 was utilised. All numerical data is presented as X/Y scatter, mean alone, or, mean ± standard error of the mean (SEM). Power values for each test where calculated post-hoc using G*Power (version 3.1, gpower.hhu.de/), based upon the effect size, group number and sample size. A p-value < 0.05 was considered statistically significant. All use of statistics is detailed in supplementary statistics Table 2.

## Electronic supplementary material

Supplementary information
