# Supplementary material for: Antibiotic-induced perturbations in microbial diversity during post-natal development alters amyloid pathology in an aged APPSWE/PS1ΔE9 murine model of Alzheimer’s disease
Source: Sci Rep. 2017 Sep 5;7:10411. doi: 10.1038/s41598-017-11047-w (PMC5585265; doi:10.1038/s41598-017-11047-w)
Supplement: Supplementary file 1 — Supplementary information [file 41598_2017_11047_MOESM1_ESM.doc]

**SUPPLEMENTARY INFORMATION**

**Antibiotic-induced perturbations in microbial diversity during post-natal development alters amyloid pathology in an aged APP_SWE_/PS1_ΔE9_ murine model of Alzheimer’s disease.**

Myles R. Minter^1, 2^, Reinhard Hinterleitner^3, 4^*, Marlies Meisel^3, 4^*, Can Zhang^5^*, Vanessa Leone^2, 3^, Xiaoqiong Zhang^1^, Paul Oyler-Castrillo^1^, Xulun Zhang^1^, Mark W. Musch^3^, Xunuo Shen^5^, Bana Jabri^3, 4^, Eugene B. Chang^2, 3^, Rudolph E. Tanzi^5^ and Sangram S. Sisodia^1, 2+^

^1^Department of Neurobiology, The University of Chicago, Chicago, IL, 60637, USA

^2^The Microbiome Center, The University of Chicago, IL, Chicago, 60637, USA

^3^Department of Medicine, The University of Chicago, IL, Chicago, 60637, USA

^4^Committee on Immunology, The University of Chicago, IL, Chicago, 60637, USA

^5^Department of Neurology, Genetics and Aging Research Unit, MassGeneral Institute for Neurodegenerative Diseases, Massachusetts General Hospital, Charlestown, MA, 02114, USA

*Denotes equal contribution.

^+^Correspondence: ssisodia@bsd.uchicago.edu


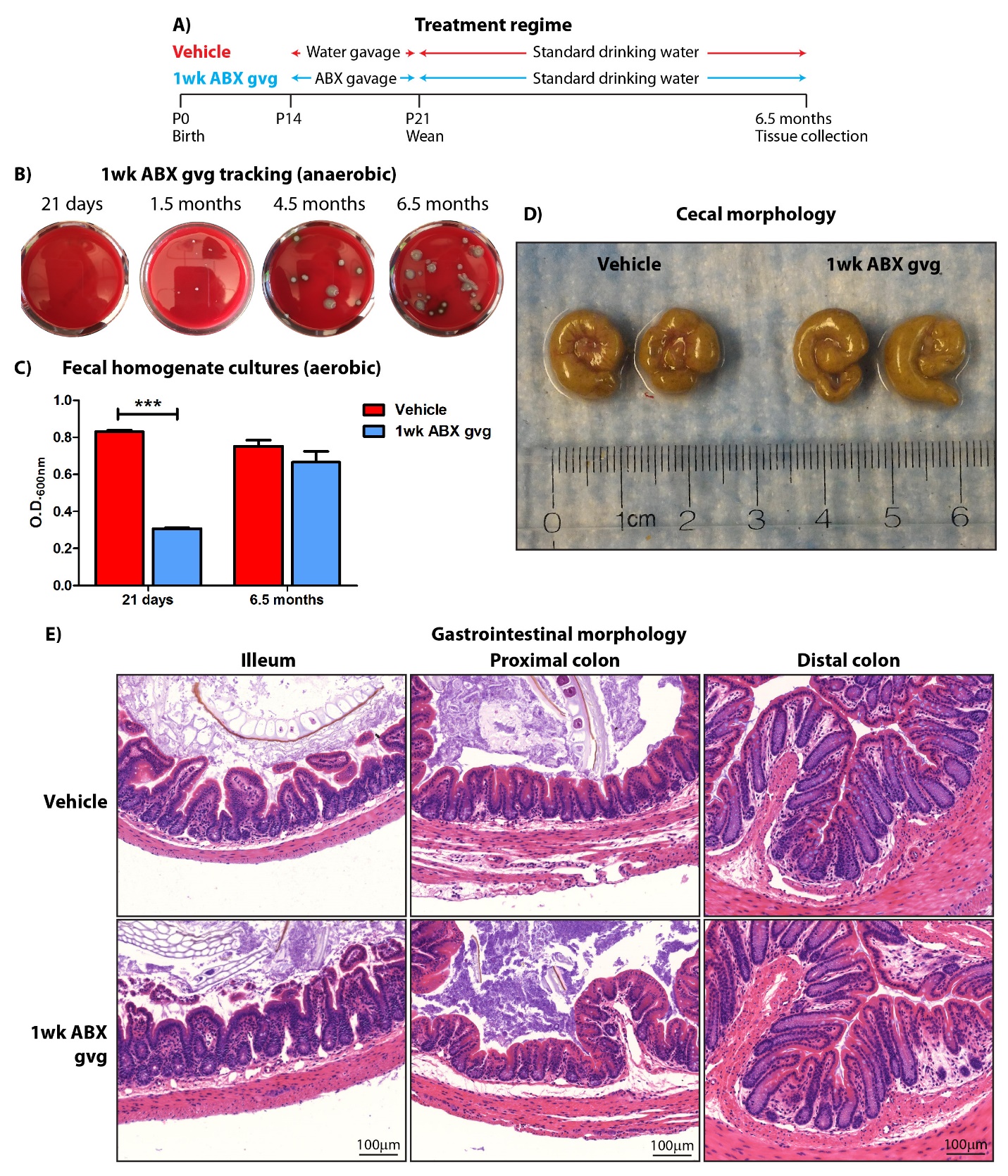


**Supplementary figure 1. Monitoring the effect of ABX treatment on gut bacterial viability and gross visceral morphology in APP_SWE_/PS1_ΔE9_ mice.**

**A)** Treatment regime schematic detailing the 1wk antibiotic (ABX) gavage (1wk ABX gvg) treatment of APP_SWE_/PS1_ΔE9_ mice used in the current study. **B)** Representative images of bacterial growth on Brucella blood medium agar plates incubated under anaerobic conditions of faecal homogenates obtained from 1 wk ABX gvg-treated APP_SWE_/PS1_ΔE9_ mice at 21 days, 1.5 months, 4.5 months and 6.5 months of age. **C)** Additional faecal homogenates from vehicle and 1wk ABX gvg-treated APP_SWE_/PS1_ΔE9_ mice prepared immediately post-gavage (21 days) or prior to tissue collection (6.5 months) were also cultured in LB media using aerobic conditions and optical density (O.D.) of cultures was measured at 600nm (*n*=6, ***p<0.05, un-paired two-tailed Student’s *t*-test). **D)** Representative images of whole caecal tissue isolated from vehicle and 1wk ABX gvg-treated APP_SWE_/PS1_ΔE9_ mice. **E)** Representative x20 magnification images of Carnoy’s-fixed hematoxylin and eosin-stained and ileum, proximal colon and distal colon cross-sections (5µm thickness) displaying intestinal crypts, smooth muscle wall and gastro-intestinal tract lumen. Data are displayed as mean ± SEM. See statistical table 1 for additional information.


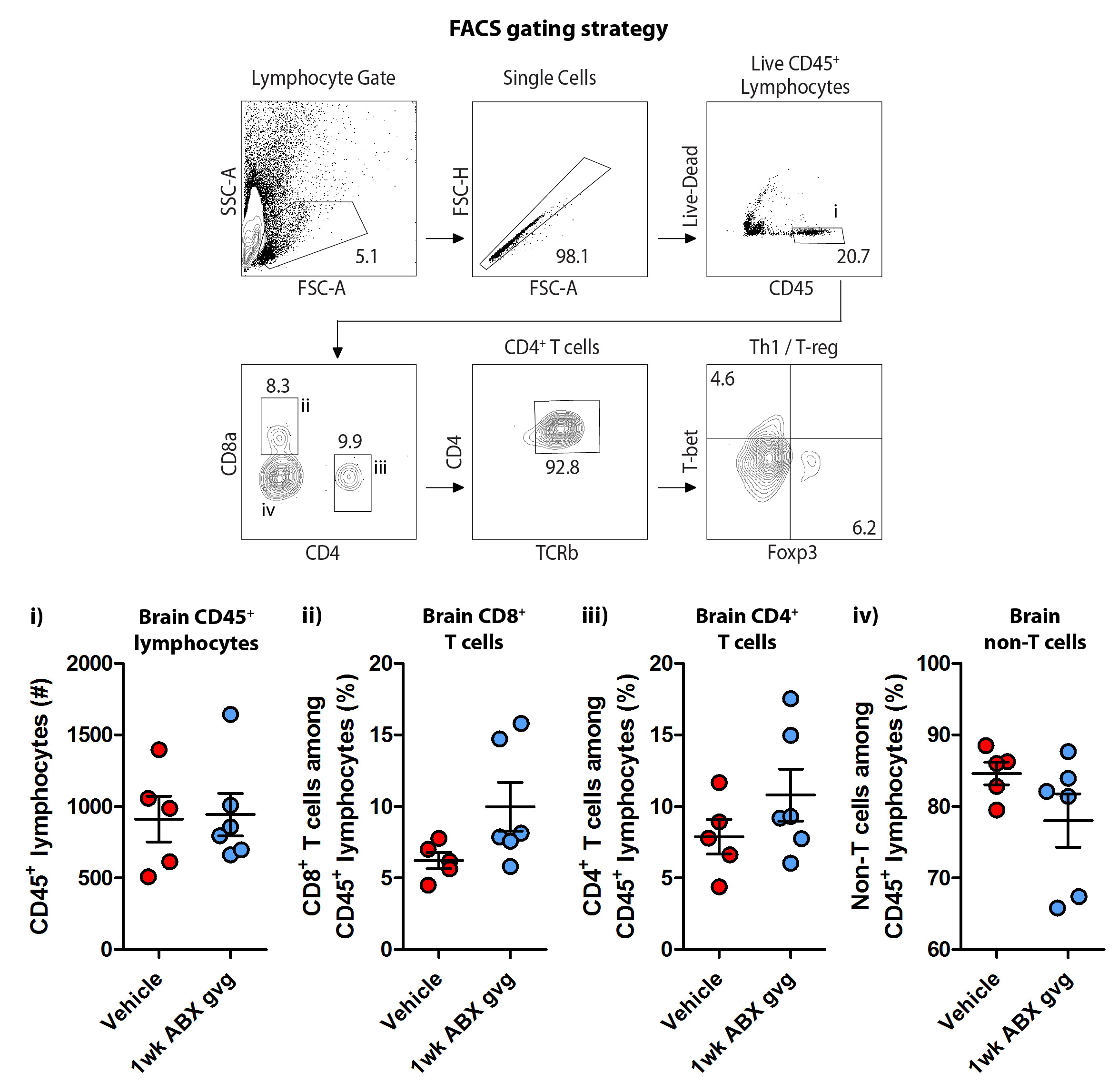


**Supplementary figure 2. General gating strategy for T-cell flow cytometry analysis and brain lymphocyte yields in APP_SWE_/PS1_ΔE9_ mice.**

The gating strategy of flow cytometry data for analysis of the intracellular expression of T-bet and Foxp3 in T cell populations of the brain. The same gating strategy is also used for flow cytometric analysis of MLN and blood tissue. Labelled inset and calculated for the entire vehicle and 1wk ABX gvg-treated APP_SWE_/PS1_ΔE9_ mouse cohort are total brain **i)** CD45^+^ lymphocyte numbers, **ii)** CD8^+^ T cell percentages, **iii)** CD4^+^ T cell percentages and **iv)** non-T cell percentages (not displayed in gating strategy). All percentages are expressed relative to total live CD45^+^ lymphocytes detected from each individual sample. All data are displayed as mean ± SEM. See statistical table 1 for additional information.


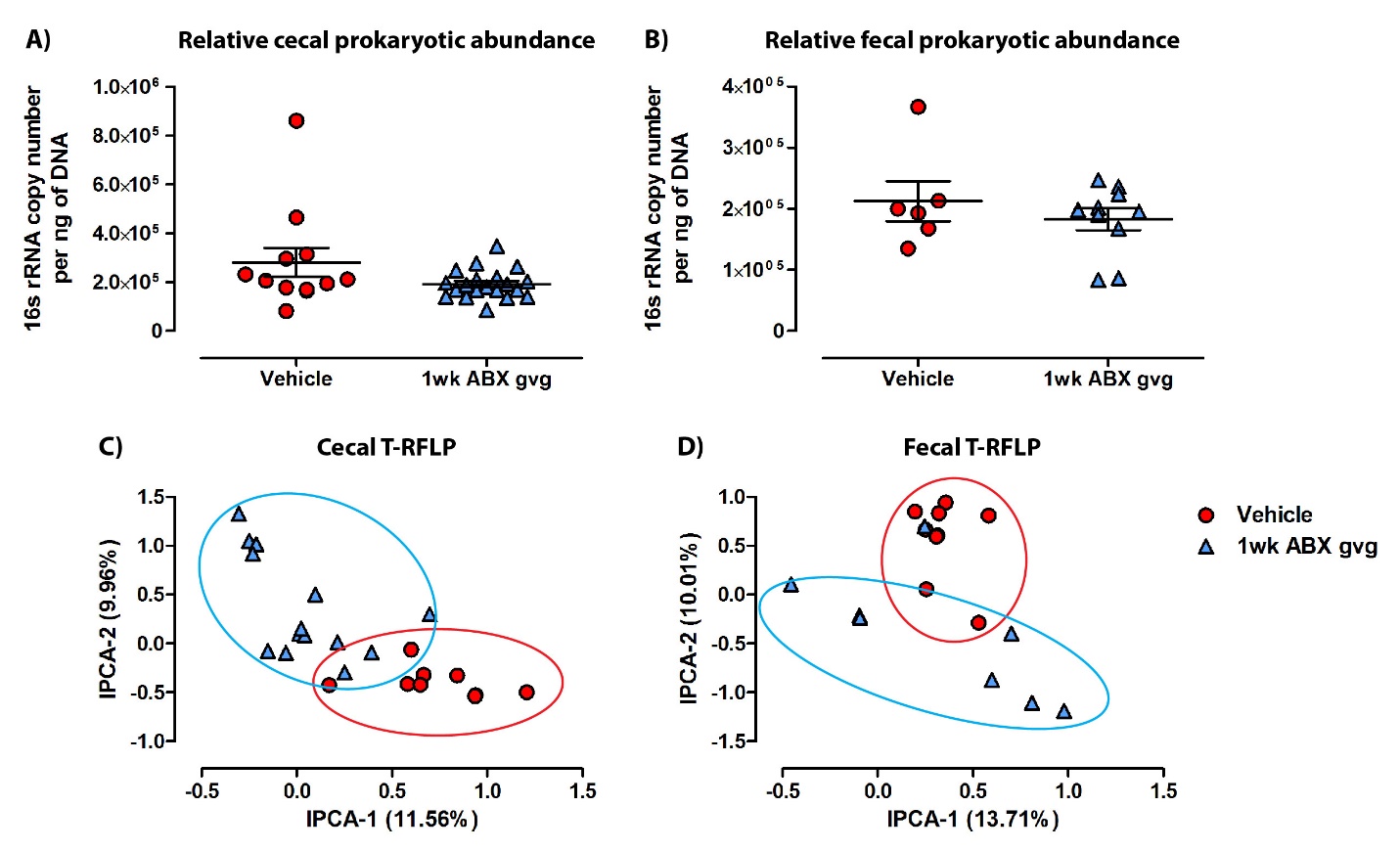


**Supplementary figure 3. Molecular analysis of the bacterial 16s rRNA gene reveals altered diversity, but not abundance, of gut bacteria in 1wk ABX gvg-treated APP_SWE_/PS1_ΔE9_ mice at the time of cull.**

**A)** Q-PCR analysis of DNA isolated from caecal matter from vehicle and 1wk ABX gvg-treated 6.5 month old APP_SWE_/PS1_ΔE9_ mice analysing 16s rRNA gene copy number (*n*=12-15). **B)** Q-PCR analysis of DNA isolated from faecal matter from vehicle and 1wk ABX gvg-treated APP_SWE_/PS1_ΔE9_ mice analysing 16s rRNA gene copy number (*n*=7-10). To obtain a 16s rRNA gene copy number value, expression levels were normalised to both DNA concentration and an amplification standard curve of a 16s rRNA gene-containing plasmid with known copy number. **C)** Principal coordinate analysis plots generated from T-RFLP analysis of the PCR-amplified 16s rRNA gene obtained from caecal contents of vehicle and 1wk ABX gvg-treated APP_SWE_/PS1_ΔE9_ mice (*n*=10-13). **D)** Principal coordinate analysis plots generated from T-RFLP analysis of the PCR-amplified 16s rRNA gene obtained from faecal contents APP_SWE_/PS1_ΔE9_ mice (*n*=8-9). The percentage of data variance explained by each IPCA for all T-RFLP analysis is displayed. Data are displayed as mean ± SEM or X/Y scatter. See statistical table 1 for additional information.


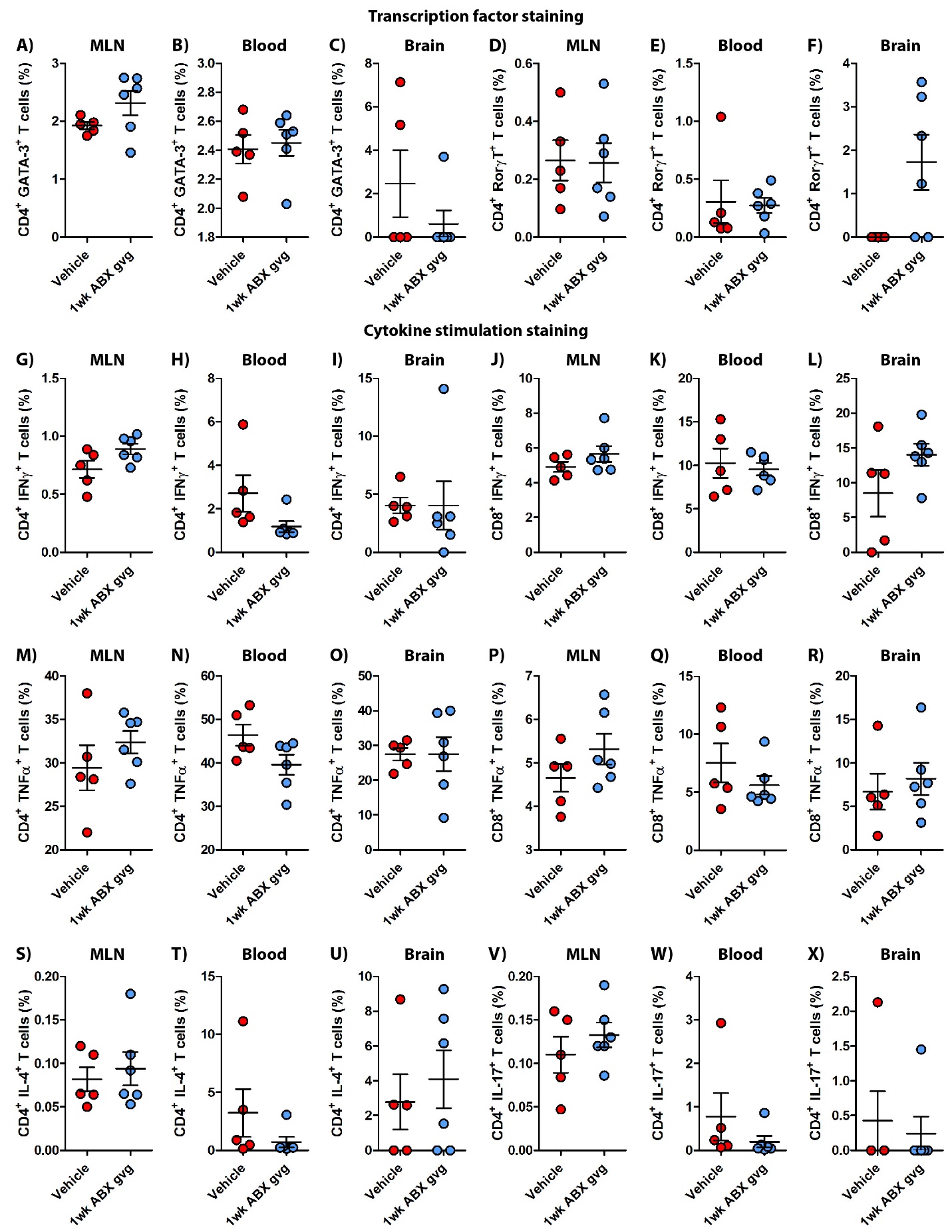


**Supplementary figure 4. Analysis of GATA-3 and RorγT intracellular expression and additional cytokine stimulation profiles in T cells detected by flow cytometry in vehicle and 1wk ABX gvg-treated APP_SWE_/PS1_ΔE9_ mice.**

Percentages of GATA-3 expressing CD4^+^ T cells were calculated within **A)** MLN, **B)** blood and **C)** brain tissues of vehicle and 1wk ABX gvg-treated APP_SWE_/PS1_ΔE9_ mice. Percentages of RorγT expressing CD4^+^ T cells were calculated within **D)** MLN, **E)** blood and **F)** brain tissues of vehicle and 1wk ABX gvg-treated APP_SWE_/PS1_ΔE9_ mice. Live cell preparations were also immunologically stimulated (see methods) to induce cytokine production and subsequently analysed by flow cytometry. Percentages of IFNγ expressing CD4^+^ T cells within the **G)** MLN, **H)** blood and **I)** brain and IFNγ expressing CD8^+^ T cells within the **J)** MLN, **K)** blood and **L)** brain were calculated from vehicle and 1wk ABX gvg-treated APP_SWE_/PS1_ΔE9_ mice. Percentages of TNFα expressing CD4^+^ T cells within the **M)** MLN, **N)** blood and **O)** brain and TNFα expressing CD8^+^ T cells within the **P)** MLN, **Q)** blood and **R)** brain were calculated from vehicle and 1wk ABX gvg-treated APP_SWE_/PS1_ΔE9_ mice. Percentages of IL-4 expressing CD4^+^ T cells were calculated within **S)** MLN, **T)** blood and **U)** brain tissues of vehicle and 1wk ABX gvg-treated APP_SWE_/PS1_ΔE9_ mice. Percentages of IL-17 expressing CD4^+^ T cells were calculated within **V)** MLN, **W)** blood and **X)** brain tissues of vehicle and 1wk ABX gvg-treated APP_SWE_/PS1_ΔE9_ mice. Quantified expression of transcription factor or cytokine expression are displayed relative to the total live CD4^+^ or CD8^+^ T cell counts (*n*=5-6). Data are displayed as mean ± SEM. See statistical table 1 for additional information.


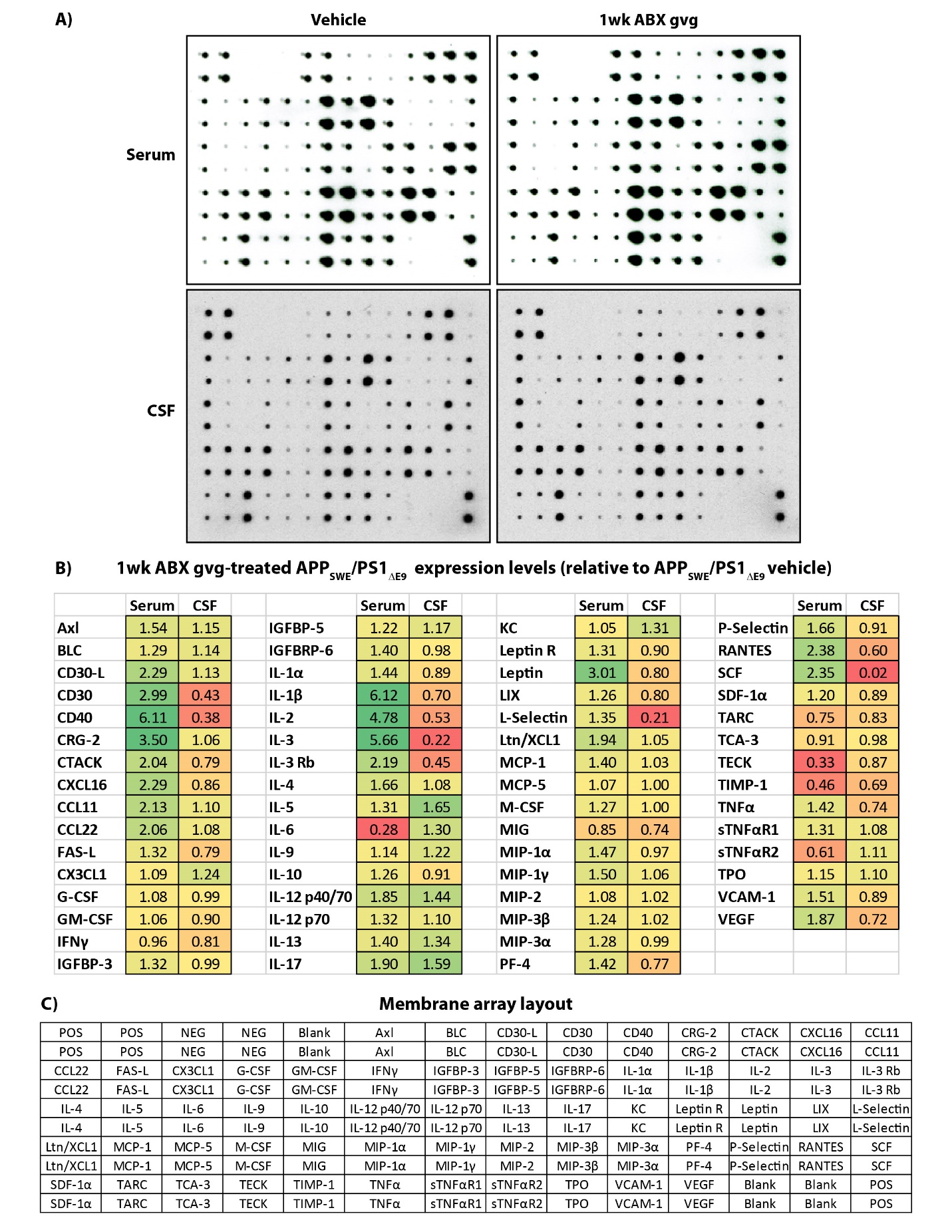


**Supplementary figure 5. Immunoblot-based array of circulating inflammatory mediators in the serum and CSF of APP_SWE_/PS1_ΔE9_ mice.**

**A)** Immunoblot-based array of inflammatory mediators in isolated serum (*n*=10 pooled sera) and CSF (*n*=10 pooled CSF) from vehicle and 1wk ABX gvg-treated 6.5 month old APP_SWE_/PS1_ΔE9_ mice. **B)** Densitometry of all 64 measured inflammatory mediator expression levels in 1wk ABX gvg-treated APP_SWE_/PS1_ΔE9_ mice relative to vehicle. Data is heat map-stratified whereby green represents up-regulated and red represents down-regulated expression relative to vehicle. **C)** The dot-printed membrane array layout is shown.


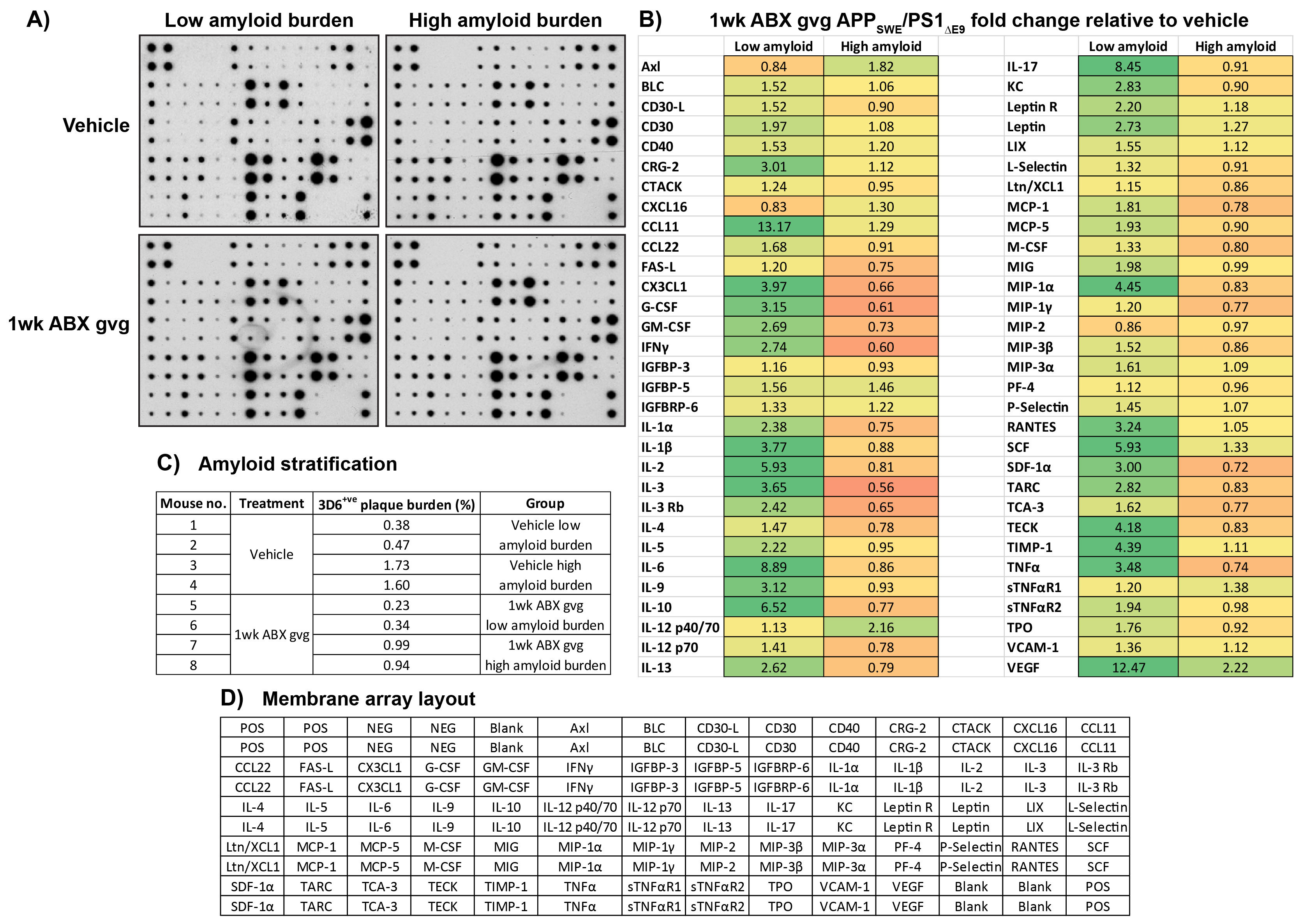


**Supplementary figure 6. Immunoblot-based array of circulating inflammatory mediators in the serum of low and high amyloid burden APP_SWE_/PS1_ΔE9_ mice.**

**A)** Immunoblot-based array of inflammatory mediators in isolated serum (*n*=2 pooled sera) from vehicle and 1wk ABX gvg-treated 6.5 month old APP_SWE_/PS1_ΔE9_ mice deemed to possess low and high amyloid burden as determined by 3D6^+ve^ stereology. **B)** Densitometry of all 64 measured inflammatory mediator expression levels in low and high amyloid burden 1wk ABX gvg-treated APP_SWE_/PS1_ΔE9_ mice relative to vehicle. Data is heat map-stratified whereby green represents up-regulated and red represents down-regulated expression relative to vehicle. **C)** Absolute percentage amyloid burden, as calculated by 3D6^+ve^ stereology, is provided for all mice stratified to the low and high amyloid groups. **D)** The dot-printed membrane array layout is shown.


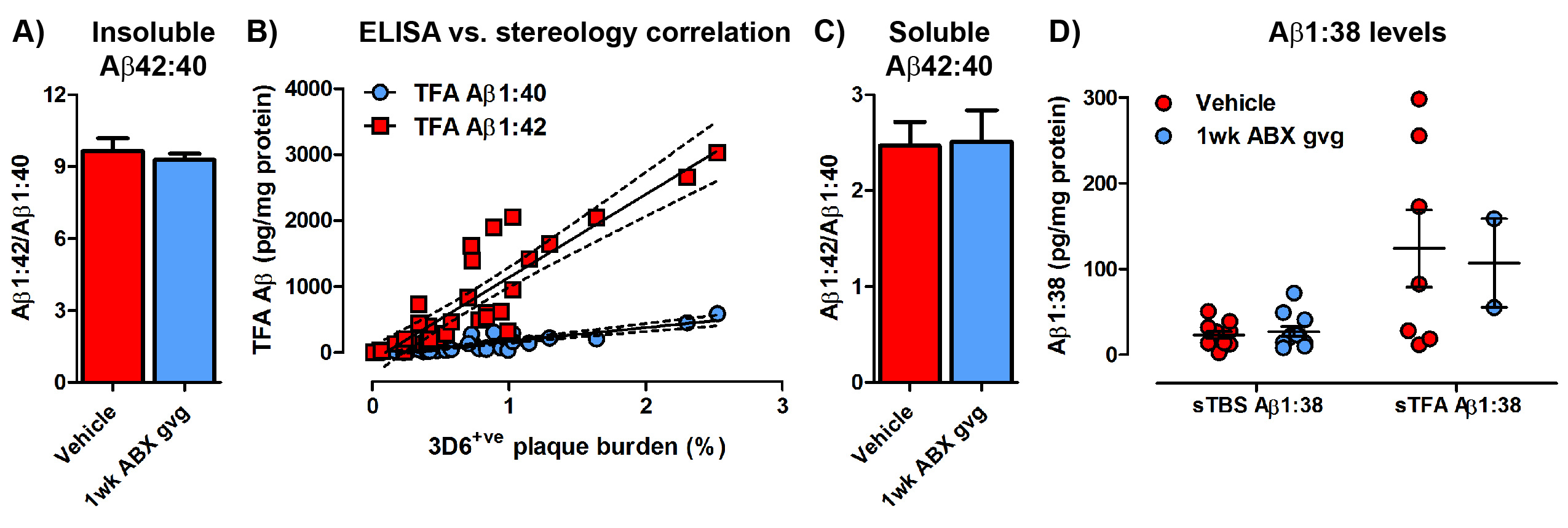


**Supplementary figure 7. Additional amyloidosis analysis in cortical and hippocampal tissue of vehicle and 1wk ABX gvg-treated APP_SWE_/PS1_ΔE9_ mice.**

**A)** Quantification of the Aβ1-42:Aβ1-40 ratio from the TFA-soluble MSD Mesoscale® data in vehicle and 1wk ABX gvg-treated APP_SWE_/PS1_ΔE9_ mice (*n*=14). **B)** Pearson’s correlation plot of sTFA Aβ1:40 and sTFA Aβ1:42 vs. 3D6^+^ immunofluorescent plaque burden (stereology) in all APP_SWE_/PS1_ΔE9_ mouse tissue analysed (*n*=28). **C)** Quantification of the Aβ1-42:Aβ1-40 ratio from the TBS-soluble MSD Mesoscale® data in vehicle and 1wk ABX gvg-treated APP_SWE_/PS1_ΔE9_ mice (*n*=14). **D)** MSD Mesoscale® analysis of TBS-soluble (sTBS) and TFA-soluble (sTFA) Aβ1:38 levels in combined cortical and hippocampal tissue from vehicle and 1wk ABX gvg-treated 6.5 month old APP_SWE_/PS1_ΔE9_ mice using anti-Aβ mAb, 4G8 (*n*=14, *p<0.05, un-paired two-tailed Student’s *t*-test). Note that Aβ1:38 was undetectable by this method in 3x vehicle sTBS, 3x 1wk ABX gvg sTBS, 7x vehicle sTFA and 12x 1wk ABX gvg sTFA samples. Data are displayed as mean ± SEM or X/Y scatter with linear line of best fit and 95% confidence interval calculated by Pearson’s linear regression analysis. See statistical table 1 for additional information.

**
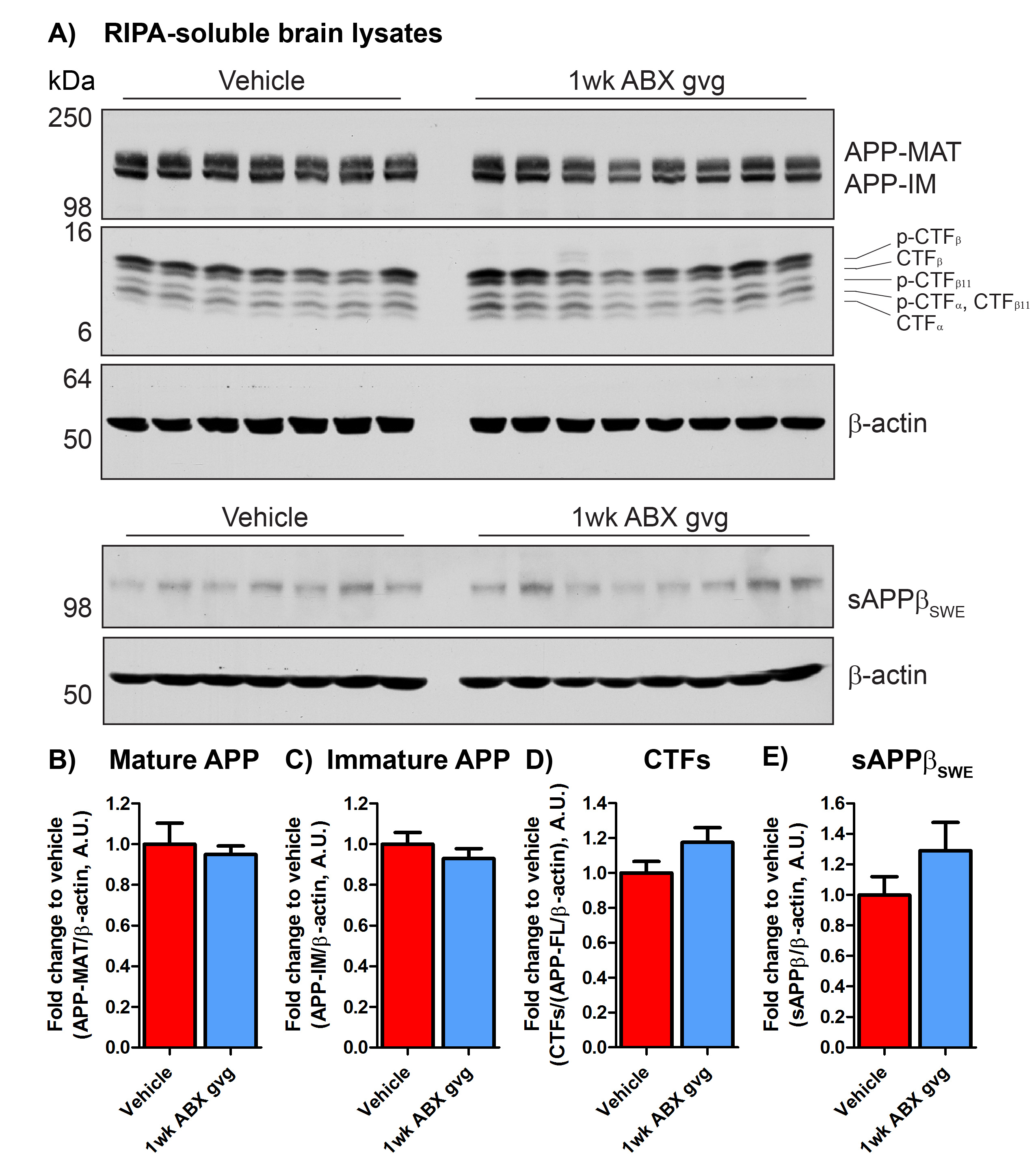
**

**Supplementary figure 8. Analysis of APP proteolytic fragments in cortical and hippocampal tissue of vehicle and 1wk ABX gvg-treated APP_SWE_/PS1_ΔE9_ mice.**

**A)** Immunoblot of mature (APP-MAT) and immature APP (APP-IM), C-terminal fragments (CTFs) of APP and the sAPPβ_SWE_ shed ectodomain in RIPA-soluble brain lysates of vehicle and 1wk ABX gvg-treated APP_SWE_/PS1_ΔE9_ mice using anti-APP mAb, C1/6.1 and an anti-sAPPβ_SWE_ mAb, 192_SWE_. APP and CTF samples were resolved on 10-16.5% w/v acrylamide tris-tricine gels and sAPPβ_SWE_ was analysed by 10% w/v acrylamide SDS-PAGE. Densitometry of **B)** APP-MAT, **C)** APP-IM, **D)** CTFs, and **E)** sAPPβ_SWE_ expression is displayed (*n*=7-8). All densitometry is expressed as a ratio of APP:β-actin, CTF:(APP: β-actin) or sAPPβ_SWE_:β-actin raw pixel intensities. Immuno-detection of β-actin was used to ascertain loading quantities. Full length blots can be viewed in supplementary figure 9. Data are displayed as mean ± SEM. See statistical table 1 for additional information.

**
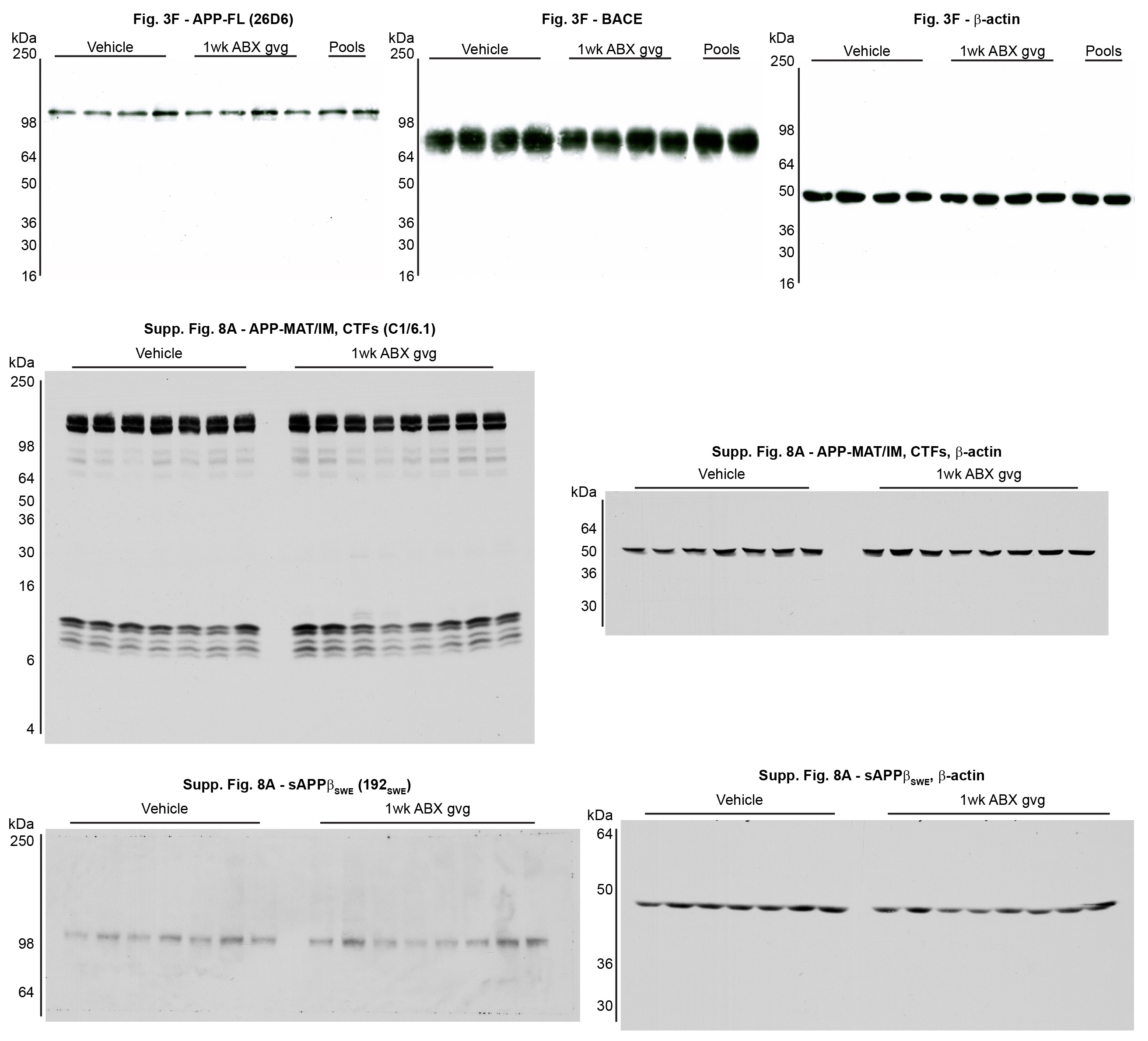
**

**Supplementary Figure 9. Full length western blots used within the article.**

Where blots are cropped and bands of interest displayed in the corresponding figures throughout this article, they are shown here in their entirety. Many blots were physically cut around the area of interest prior to antibody incubation and are presented as such.

**Supplementary table 1: Complete taxonomy assignment percentages from Illumina®** MiSeq amplicon sequencing data of the V4-V5 variable region of the 16s rRNA gene.

| **Taxonomy** | **Proportion of total OTU reads (%)** | |
| --- | --- | --- |
|  | **Treatment** | |
|  | **Vehicle** | **1wk ABX gvg** |
| k__Archaea;p__Euryarchaeota;c__Methanomicrobia;o__Methanosarcinales;f__Methanosarcinaceae;g__Methanosarcina | 0.0000000 | 0.0000000 |
| k__Bacteria;p__Acidobacteria;c__Solibacteres;o__Solibacterales;f__Solibacteraceae;g__Candidatus Solibacter | 0.0000000 | 0.0009613 |
| k__Bacteria;p__Actinobacteria;c__Actinobacteria;o__Actinomycetales;Other;Other | 0.0000190 | 0.0002068 |
| k__Bacteria;p__Actinobacteria;c__Actinobacteria;o__Actinomycetales;f__Brevibacteriaceae;g__Brevibacterium | 0.0000379 | 0.0000000 |
| k__Bacteria;p__Actinobacteria;c__Actinobacteria;o__Actinomycetales;f__Corynebacteriaceae;g__Corynebacterium | 0.0007777 | 0.0000495 |
| k__Bacteria;p__Actinobacteria;c__Actinobacteria;o__Actinomycetales;f__Dermabacteraceae;g__Brachybacterium | 0.0000000 | 0.0000000 |
| k__Bacteria;p__Actinobacteria;c__Actinobacteria;o__Actinomycetales;f__Gordoniaceae;g__ | 0.0000000 | 0.0000000 |
| k__Bacteria;p__Actinobacteria;c__Actinobacteria;o__Actinomycetales;f__Microbacteriaceae;Other | 0.0002276 | 0.0002231 |
| k__Bacteria;p__Actinobacteria;c__Actinobacteria;o__Actinomycetales;f__Microbacteriaceae;g__Microbacterium | 0.0000379 | 0.0000000 |
| k__Bacteria;p__Actinobacteria;c__Actinobacteria;o__Actinomycetales;f__Microbacteriaceae;g__Salinibacterium | 0.0000000 | 0.0002015 |
| k__Bacteria;p__Actinobacteria;c__Actinobacteria;o__Actinomycetales;f__Micrococcaceae;g__Arthrobacter | 0.0000190 | 0.0002512 |
| k__Bacteria;p__Actinobacteria;c__Actinobacteria;o__Actinomycetales;f__Micrococcaceae;g__Kocuria | 0.0000379 | 0.0000000 |
| k__Bacteria;p__Actinobacteria;c__Actinobacteria;o__Actinomycetales;f__Nocardiaceae;g__Rhodococcus | 0.0000569 | 0.0000889 |
| k__Bacteria;p__Actinobacteria;c__Actinobacteria;o__Actinomycetales;f__Nocardioidaceae;g__ | 0.0000000 | 0.0001735 |
| k__Bacteria;p__Actinobacteria;c__Actinobacteria;o__Actinomycetales;f__Nocardiopsaceae;Other | 0.0000000 | 0.0000000 |
| k__Bacteria;p__Actinobacteria;c__Actinobacteria;o__Actinomycetales;f__Promicromonosporaceae;g__Cellulosimicrobium | 0.0003793 | 0.0000000 |
| k__Bacteria;p__Actinobacteria;c__Actinobacteria;o__Actinomycetales;f__Propionibacteriaceae;g__Propionibacterium | 0.0000000 | 0.0000000 |
| k__Bacteria;p__Actinobacteria;c__Actinobacteria;o__Actinomycetales;f__Streptomycetaceae;Other | 0.0000000 | 0.0000000 |
| k__Bacteria;p__Actinobacteria;c__Actinobacteria;o__Actinomycetales;f__Yaniellaceae;g__Yaniella | 0.0000000 | 0.0000000 |
| k__Bacteria;p__Actinobacteria;c__Actinobacteria;o__Bifidobacteriales;Other;Other | 0.0000190 | 0.0021053 |
| k__Bacteria;p__Actinobacteria;c__Actinobacteria;o__Bifidobacteriales;f__Bifidobacteriaceae;g__Bifidobacterium | 0.0823947 | 1.2671873 |
| k__Bacteria;p__Actinobacteria;c__Thermoleophilia;o__Solirubrobacterales;Other;Other | 0.0000190 | 0.0002867 |
| k__Bacteria;p__Bacteroidetes;Other;Other;Other;Other | 0.0990671 | 0.1027797 |
| k__Bacteria;p__Bacteroidetes;c__Bacteroidia;Other;Other;Other | 0.0005121 | 0.0000696 |
| k__Bacteria;p__Bacteroidetes;c__Bacteroidia;o__Bacteroidales;Other;Other | 7.4351540 | 0.6607606 |
| k__Bacteria;p__Bacteroidetes;c__Bacteroidia;o__Bacteroidales;f__;g__ | 4.7711157 | 1.9082582 |
| k__Bacteria;p__Bacteroidetes;c__Bacteroidia;o__Bacteroidales;f__Bacteroidaceae;Other | 0.0239749 | 0.0115878 |
| k__Bacteria;p__Bacteroidetes;c__Bacteroidia;o__Bacteroidales;f__Bacteroidaceae;g__Bacteroides | 4.7273388 | 6.3776119 |
| k__Bacteria;p__Bacteroidetes;c__Bacteroidia;o__Bacteroidales;f__Porphyromonadaceae;Other | 0.0073594 | 0.0005245 |
| k__Bacteria;p__Bacteroidetes;c__Bacteroidia;o__Bacteroidales;f__Porphyromonadaceae;g__Parabacteroides | 3.8229698 | 0.6065396 |
| k__Bacteria;p__Bacteroidetes;c__Bacteroidia;o__Bacteroidales;f__Prevotellaceae;Other | 0.0382764 | 0.0034617 |
| k__Bacteria;p__Bacteroidetes;c__Bacteroidia;o__Bacteroidales;f__Prevotellaceae;g__Prevotella | 2.8286190 | 0.2563062 |
| k__Bacteria;p__Bacteroidetes;c__Bacteroidia;o__Bacteroidales;f__Rikenellaceae;Other | 0.1416301 | 0.0054800 |
| k__Bacteria;p__Bacteroidetes;c__Bacteroidia;o__Bacteroidales;f__Rikenellaceae;g__ | 2.7989918 | 1.4237842 |
| k__Bacteria;p__Bacteroidetes;c__Bacteroidia;o__Bacteroidales;f__Rikenellaceae;g__AF12 | 0.2013776 | 0.0000625 |
| k__Bacteria;p__Bacteroidetes;c__Bacteroidia;o__Bacteroidales;f__Rikenellaceae;g__Rikenella | 0.0000948 | 0.0000387 |
| k__Bacteria;p__Bacteroidetes;c__Bacteroidia;o__Bacteroidales;f__S24-7;g__ | 27.8169730 | 15.7017975 |
| k__Bacteria;p__Bacteroidetes;c__Bacteroidia;o__Bacteroidales;f__[Odoribacteraceae];Other | 0.0001707 | 0.0000000 |
| k__Bacteria;p__Bacteroidetes;c__Bacteroidia;o__Bacteroidales;f__[Odoribacteraceae];g__Odoribacter | 1.5111195 | 0.0011029 |
| k__Bacteria;p__Bacteroidetes;c__Bacteroidia;o__Bacteroidales;f__[Paraprevotellaceae];Other | 0.0003224 | 0.0000000 |
| k__Bacteria;p__Bacteroidetes;c__Bacteroidia;o__Bacteroidales;f__[Paraprevotellaceae];g__Paraprevotella | 0.4470443 | 0.0062225 |
| k__Bacteria;p__Bacteroidetes;c__Bacteroidia;o__Bacteroidales;f__[Paraprevotellaceae];g__[Prevotella] | 0.0002466 | 0.0000000 |
| k__Bacteria;p__Chloroflexi;c__Chloroflexi;o__;f__;g__ | 0.0000190 | 0.0000975 |
| k__Bacteria;p__Cyanobacteria;Other;Other;Other;Other | 0.0007208 | 0.0000602 |
| k__Bacteria;p__Cyanobacteria;c__4C0d-2;Other;Other;Other | 0.0002086 | 0.0000301 |
| k__Bacteria;p__Cyanobacteria;c__4C0d-2;o__YS2;f__;g__ | 0.4109682 | 0.0054475 |
| k__Bacteria;p__Cyanobacteria;c__Chloroplast;o__Streptophyta;f__;g__ | 0.0022002 | 0.0012595 |
| k__Bacteria;p__Cyanobacteria;c__Synechococcophycideae;o__Pseudanabaenales;f__Pseudanabaenaceae;Other | 0.0001517 | 0.0000000 |
| k__Bacteria;p__Cyanobacteria;c__Synechococcophycideae;o__Pseudanabaenales;f__Pseudanabaenaceae;g__ | 0.0000000 | 0.0000000 |
| k__Bacteria;p__Cyanobacteria;c__Synechococcophycideae;o__Pseudanabaenales;f__Pseudanabaenaceae;g__Halomicronema | 0.0000190 | 0.0000000 |
| k__Bacteria;p__Deferribacteres;c__Deferribacteres;o__Deferribacterales;f__Deferribacteraceae;g__Mucispirillum | 0.8609525 | 0.0010972 |
| k__Bacteria;p__Firmicutes;Other;Other;Other;Other | 0.0996741 | 0.7759790 |
| k__Bacteria;p__Firmicutes;c__Bacilli;Other;Other;Other | 0.0086681 | 0.4007681 |
| k__Bacteria;p__Firmicutes;c__Bacilli;o__Bacillales;Other;Other | 0.0000569 | 0.0000637 |
| k__Bacteria;p__Firmicutes;c__Bacilli;o__Bacillales;f__Bacillaceae;Other | 0.0000379 | 0.0000000 |
| k__Bacteria;p__Firmicutes;c__Bacilli;o__Bacillales;f__Bacillaceae;g__Geobacillus | 0.0001138 | 0.0000998 |
| k__Bacteria;p__Firmicutes;c__Bacilli;o__Bacillales;f__Paenibacillaceae;g__Paenibacillus | 0.0000000 | 0.0000000 |
| k__Bacteria;p__Firmicutes;c__Bacilli;o__Bacillales;f__Planococcaceae;Other | 0.0000948 | 0.0000000 |
| k__Bacteria;p__Firmicutes;c__Bacilli;o__Bacillales;f__Planococcaceae;g__Sporosarcina | 0.0002086 | 0.0000313 |
| k__Bacteria;p__Firmicutes;c__Bacilli;o__Bacillales;f__Staphylococcaceae;Other | 0.0000948 | 0.0000000 |
| k__Bacteria;p__Firmicutes;c__Bacilli;o__Bacillales;f__Staphylococcaceae;g__Jeotgalicoccus | 0.0002655 | 0.0001590 |
| k__Bacteria;p__Firmicutes;c__Bacilli;o__Bacillales;f__Staphylococcaceae;g__Staphylococcus | 0.0027123 | 0.0012801 |
| k__Bacteria;p__Firmicutes;c__Bacilli;o__Lactobacillales;Other;Other | 0.0149464 | 0.0484919 |
| k__Bacteria;p__Firmicutes;c__Bacilli;o__Lactobacillales;f__Aerococcaceae;g__ | 0.0000379 | 0.0035139 |
| k__Bacteria;p__Firmicutes;c__Bacilli;o__Lactobacillales;f__Aerococcaceae;g__Aerococcus | 0.0004742 | 0.0000000 |
| k__Bacteria;p__Firmicutes;c__Bacilli;o__Lactobacillales;f__Enterococcaceae;Other | 0.0011191 | 0.0014378 |
| k__Bacteria;p__Firmicutes;c__Bacilli;o__Lactobacillales;f__Enterococcaceae;g__Enterococcus | 0.3312100 | 0.1441661 |
| k__Bacteria;p__Firmicutes;c__Bacilli;o__Lactobacillales;f__Lactobacillaceae;Other | 0.0970945 | 0.4103542 |
| k__Bacteria;p__Firmicutes;c__Bacilli;o__Lactobacillales;f__Lactobacillaceae;g__ | 2.3423500 | 5.7949858 |
| k__Bacteria;p__Firmicutes;c__Bacilli;o__Lactobacillales;f__Lactobacillaceae;g__Lactobacillus | 0.3985066 | 0.4362281 |
| k__Bacteria;p__Firmicutes;c__Bacilli;o__Lactobacillales;f__Streptococcaceae;Other | 0.0000000 | 0.0000000 |
| k__Bacteria;p__Firmicutes;c__Bacilli;o__Lactobacillales;f__Streptococcaceae;g__Streptococcus | 0.0409887 | 0.0076637 |
| k__Bacteria;p__Firmicutes;c__Bacilli;o__Turicibacterales;f__Turicibacteraceae;g__Turicibacter | 0.1156826 | 3.1452182 |
| k__Bacteria;p__Firmicutes;c__Clostridia;Other;Other;Other | 1.1150218 | 0.9471276 |
| k__Bacteria;p__Firmicutes;c__Clostridia;o__;f__;g__ | 0.1047194 | 0.1847214 |
| k__Bacteria;p__Firmicutes;c__Clostridia;o__Clostridiales;Other;Other | 0.1819739 | 0.3510034 |
| k__Bacteria;p__Firmicutes;c__Clostridia;o__Clostridiales;f__;g__ | 0.5265560 | 0.4689172 |
| k__Bacteria;p__Firmicutes;c__Clostridia;o__Clostridiales;f__Christensenellaceae;g__ | 0.0047039 | 0.0000000 |
| k__Bacteria;p__Firmicutes;c__Clostridia;o__Clostridiales;f__Clostridiaceae;Other | 0.0003793 | 0.0005628 |
| k__Bacteria;p__Firmicutes;c__Clostridia;o__Clostridiales;f__Clostridiaceae;g__ | 0.1415353 | 0.0139589 |
| k__Bacteria;p__Firmicutes;c__Clostridia;o__Clostridiales;f__Clostridiaceae;g__Clostridium | 0.0351278 | 0.0513126 |
| k__Bacteria;p__Firmicutes;c__Clostridia;o__Clostridiales;f__Dehalobacteriaceae;g__Dehalobacterium | 0.2422905 | 0.0319424 |
| k__Bacteria;p__Firmicutes;c__Clostridia;o__Clostridiales;f__Eubacteriaceae;g__Anaerofustis | 0.0076249 | 0.0000000 |
| k__Bacteria;p__Firmicutes;c__Clostridia;o__Clostridiales;f__Lachnospiraceae;Other | 5.1033120 | 12.2702393 |
| k__Bacteria;p__Firmicutes;c__Clostridia;o__Clostridiales;f__Lachnospiraceae;g__ | 11.3702974 | 28.8533800 |
| k__Bacteria;p__Firmicutes;c__Clostridia;o__Clostridiales;f__Lachnospiraceae;g__Anaerostipes | 0.0971893 | 0.0000000 |
| k__Bacteria;p__Firmicutes;c__Clostridia;o__Clostridiales;f__Lachnospiraceae;g__Blautia | 0.0000190 | 0.0064697 |
| k__Bacteria;p__Firmicutes;c__Clostridia;o__Clostridiales;f__Lachnospiraceae;g__Dorea | 0.2276666 | 0.1289350 |
| k__Bacteria;p__Firmicutes;c__Clostridia;o__Clostridiales;f__Lachnospiraceae;g__[Ruminococcus] | 0.5957114 | 0.5792316 |
| k__Bacteria;p__Firmicutes;c__Clostridia;o__Clostridiales;f__Peptostreptococcaceae;Other | 0.0007018 | 0.0121436 |
| k__Bacteria;p__Firmicutes;c__Clostridia;o__Clostridiales;f__Peptostreptococcaceae;g__ | 0.0498845 | 0.4757975 |
| k__Bacteria;p__Firmicutes;c__Clostridia;o__Clostridiales;f__Ruminococcaceae;Other | 1.0484649 | 1.6251296 |
| k__Bacteria;p__Firmicutes;c__Clostridia;o__Clostridiales;f__Ruminococcaceae;g__ | 1.5980474 | 0.3997569 |
| k__Bacteria;p__Firmicutes;c__Clostridia;o__Clostridiales;f__Ruminococcaceae;g__Faecalibacterium | 0.0000569 | 0.0000000 |
| k__Bacteria;p__Firmicutes;c__Clostridia;o__Clostridiales;f__Ruminococcaceae;g__Oscillospira | 4.8275250 | 7.4772734 |
| k__Bacteria;p__Firmicutes;c__Clostridia;o__Clostridiales;f__Ruminococcaceae;g__Ruminococcus | 1.3334322 | 2.3980824 |
| k__Bacteria;p__Firmicutes;c__Clostridia;o__Coriobacteriales;f__Coriobacteriaceae;Other | 0.0099390 | 0.0081406 |
| k__Bacteria;p__Firmicutes;c__Clostridia;o__Coriobacteriales;f__Coriobacteriaceae;g__ | 0.0046850 | 0.0382923 |
| k__Bacteria;p__Firmicutes;c__Clostridia;o__Coriobacteriales;f__Coriobacteriaceae;g__Adlercreutzia | 0.1044349 | 0.0209069 |
| k__Bacteria;p__Firmicutes;c__Erysipelotrichi;o__Erysipelotrichales;Other;Other | 0.0010432 | 0.0000313 |
| k__Bacteria;p__Firmicutes;c__Erysipelotrichi;o__Erysipelotrichales;f__Erysipelotrichaceae;Other | 0.0045332 | 0.0004038 |
| k__Bacteria;p__Firmicutes;c__Erysipelotrichi;o__Erysipelotrichales;f__Erysipelotrichaceae;g__ | 0.2426698 | 0.3875126 |
| k__Bacteria;p__Firmicutes;c__Erysipelotrichi;o__Erysipelotrichales;f__Erysipelotrichaceae;g__Allobaculum | 0.9776783 | 0.3775423 |
| k__Bacteria;p__Firmicutes;c__Erysipelotrichi;o__Erysipelotrichales;f__[Coprobacillaceae];Other | 0.0166534 | 0.0019449 |
| k__Bacteria;p__Firmicutes;c__Erysipelotrichi;o__Erysipelotrichales;f__[Coprobacillaceae];g__ | 0.5625562 | 0.6070419 |
| k__Bacteria;p__Firmicutes;c__Erysipelotrichi;o__Erysipelotrichales;f__[Coprobacillaceae];g__Coprobacillus | 1.1110576 | 0.0637717 |
| k__Bacteria;p__Proteobacteria;Other;Other;Other;Other | 0.0066196 | 0.0057191 |
| k__Bacteria;p__Proteobacteria;c__Alphaproteobacteria;Other;Other;Other | 0.0044005 | 0.0001723 |
| k__Bacteria;p__Proteobacteria;c__Alphaproteobacteria;o__;f__;g__ | 0.0116081 | 0.0003369 |
| k__Bacteria;p__Proteobacteria;c__Alphaproteobacteria;o__Caulobacterales;f__Caulobacteraceae;g__ | 0.0000759 | 0.0001571 |
| k__Bacteria;p__Proteobacteria;c__Alphaproteobacteria;o__Caulobacterales;f__Caulobacteraceae;g__Brevundimonas | 0.0000000 | 0.0000902 |
| k__Bacteria;p__Proteobacteria;c__Alphaproteobacteria;o__Caulobacterales;f__Caulobacteraceae;g__Caulobacter | 0.0000569 | 0.0000000 |
| k__Bacteria;p__Proteobacteria;c__Alphaproteobacteria;o__RF32;f__;g__ | 0.3223711 | 0.0016790 |
| k__Bacteria;p__Proteobacteria;c__Alphaproteobacteria;o__Rhizobiales;Other;Other | 0.0000000 | 0.0004674 |
| k__Bacteria;p__Proteobacteria;c__Alphaproteobacteria;o__Rhizobiales;f__Bradyrhizobiaceae;Other | 0.0000000 | 0.0000000 |
| k__Bacteria;p__Proteobacteria;c__Alphaproteobacteria;o__Rhizobiales;f__Bradyrhizobiaceae;g__Bradyrhizobium | 0.0000000 | 0.0004240 |
| k__Bacteria;p__Proteobacteria;c__Alphaproteobacteria;o__Rhizobiales;f__Brucellaceae;g__Ochrobactrum | 0.0015553 | 0.0000000 |
| k__Bacteria;p__Proteobacteria;c__Alphaproteobacteria;o__Rhizobiales;f__Methylobacteriaceae;g__ | 0.0004742 | 0.0000000 |
| k__Bacteria;p__Proteobacteria;c__Alphaproteobacteria;o__Rhizobiales;f__Methylobacteriaceae;g__Methylobacterium | 0.0001897 | 0.0000000 |
| k__Bacteria;p__Proteobacteria;c__Alphaproteobacteria;o__Rhizobiales;f__Methylocystaceae;g__ | 0.0001897 | 0.0000000 |
| k__Bacteria;p__Proteobacteria;c__Alphaproteobacteria;o__Rhizobiales;f__Phyllobacteriaceae;Other | 0.0000379 | 0.0000000 |
| k__Bacteria;p__Proteobacteria;c__Alphaproteobacteria;o__Rhizobiales;f__Phyllobacteriaceae;g__Phyllobacterium | 0.0000948 | 0.0000000 |
| k__Bacteria;p__Proteobacteria;c__Alphaproteobacteria;o__Rhizobiales;f__Xanthobacteraceae;g__Azorhizobium | 0.0000948 | 0.0000000 |
| k__Bacteria;p__Proteobacteria;c__Alphaproteobacteria;o__Rhodospirillales;f__Acetobacteraceae;g__ | 0.0001138 | 0.0000000 |
| k__Bacteria;p__Proteobacteria;c__Alphaproteobacteria;o__Rickettsiales;f__mitochondria;g__ | 0.0000000 | 0.0000000 |
| k__Bacteria;p__Proteobacteria;c__Alphaproteobacteria;o__Sphingomonadales;f__Erythrobacteraceae;Other | 0.0000000 | 0.0000000 |
| k__Bacteria;p__Proteobacteria;c__Alphaproteobacteria;o__Sphingomonadales;f__Sphingomonadaceae;Other | 0.0000759 | 0.0004559 |
| k__Bacteria;p__Proteobacteria;c__Alphaproteobacteria;o__Sphingomonadales;f__Sphingomonadaceae;g__Sphingomonas | 0.0027882 | 0.0001906 |
| k__Bacteria;p__Proteobacteria;c__Betaproteobacteria;Other;Other;Other | 0.0024278 | 0.1073681 |
| k__Bacteria;p__Proteobacteria;c__Betaproteobacteria;o__Burkholderiales;Other;Other | 0.2462926 | 0.1234001 |
| k__Bacteria;p__Proteobacteria;c__Betaproteobacteria;o__Burkholderiales;f__Alcaligenaceae;g__Sutterella | 0.0443270 | 0.0330833 |
| k__Bacteria;p__Proteobacteria;c__Betaproteobacteria;o__Burkholderiales;f__Comamonadaceae;Other | 0.0001517 | 0.0006603 |
| k__Bacteria;p__Proteobacteria;c__Betaproteobacteria;o__Burkholderiales;f__Comamonadaceae;g__Hydrogenophaga | 0.0000000 | 0.0000000 |
| k__Bacteria;p__Proteobacteria;c__Betaproteobacteria;o__Burkholderiales;f__Oxalobacteraceae;Other | 0.0013088 | 0.0003635 |
| k__Bacteria;p__Proteobacteria;c__Betaproteobacteria;o__Burkholderiales;f__Oxalobacteraceae;g__Herbaspirillum | 0.0000379 | 0.0000000 |
| k__Bacteria;p__Proteobacteria;c__Betaproteobacteria;o__Burkholderiales;f__Oxalobacteraceae;g__Oxalobacter | 0.0126703 | 0.0014445 |
| k__Bacteria;p__Proteobacteria;c__Betaproteobacteria;o__Burkholderiales;f__Oxalobacteraceae;g__Ralstonia | 0.0000569 | 0.0000000 |
| k__Bacteria;p__Proteobacteria;c__Betaproteobacteria;o__Neisseriales;f__Neisseriaceae;g__Neisseria | 0.0000000 | 0.0000000 |
| k__Bacteria;p__Proteobacteria;c__Deltaproteobacteria;Other;Other;Other | 0.0001517 | 0.0005343 |
| k__Bacteria;p__Proteobacteria;c__Deltaproteobacteria;o__Desulfovibrionales;Other;Other | 0.0051971 | 0.0026784 |
| k__Bacteria;p__Proteobacteria;c__Deltaproteobacteria;o__Desulfovibrionales;f__Desulfohalobiaceae;g__ | 0.0001138 | 0.0000000 |
| k__Bacteria;p__Proteobacteria;c__Deltaproteobacteria;o__Desulfovibrionales;f__Desulfovibrionaceae;Other | 0.0211108 | 0.0002803 |
| k__Bacteria;p__Proteobacteria;c__Deltaproteobacteria;o__Desulfovibrionales;f__Desulfovibrionaceae;g__Bilophila | 0.2951528 | 0.0004146 |
| k__Bacteria;p__Proteobacteria;c__Deltaproteobacteria;o__Desulfovibrionales;f__Desulfovibrionaceae;g__Desulfovibrio | 0.7463321 | 0.1518813 |
| k__Bacteria;p__Proteobacteria;c__Deltaproteobacteria;o__Myxococcales;Other;Other | 0.0000000 | 0.0003953 |
| k__Bacteria;p__Proteobacteria;c__Deltaproteobacteria;o__Myxococcales;f__;g__ | 0.0000759 | 0.0002700 |
| k__Bacteria;p__Proteobacteria;c__Epsilonproteobacteria;Other;Other;Other | 0.0001138 | 0.0000000 |
| k__Bacteria;p__Proteobacteria;c__Epsilonproteobacteria;o__Campylobacterales;Other;Other | 0.0000759 | 0.0000000 |
| k__Bacteria;p__Proteobacteria;c__Epsilonproteobacteria;o__Campylobacterales;f__Helicobacteraceae;Other | 0.0069800 | 0.0000430 |
| k__Bacteria;p__Proteobacteria;c__Epsilonproteobacteria;o__Campylobacterales;f__Helicobacteraceae;g__Flexispira | 0.0000379 | 0.0000000 |
| k__Bacteria;p__Proteobacteria;c__Epsilonproteobacteria;o__Campylobacterales;f__Helicobacteraceae;g__Helicobacter | 2.8778776 | 0.0037301 |
| k__Bacteria;p__Proteobacteria;c__Gammaproteobacteria;Other;Other;Other | 0.0001707 | 0.0020880 |
| k__Bacteria;p__Proteobacteria;c__Gammaproteobacteria;o__Alteromonadales;f__Pseudoalteromonadaceae;g__Pseudoalteromonas | 0.0000190 | 0.0000000 |
| k__Bacteria;p__Proteobacteria;c__Gammaproteobacteria;o__Enterobacteriales;f__Enterobacteriaceae;Other | 0.0009294 | 0.0015702 |
| k__Bacteria;p__Proteobacteria;c__Gammaproteobacteria;o__Enterobacteriales;f__Enterobacteriaceae;g__Erwinia | 0.0000000 | 0.0000000 |
| k__Bacteria;p__Proteobacteria;c__Gammaproteobacteria;o__Enterobacteriales;f__Enterobacteriaceae;g__Escherichia | 0.0084216 | 0.0272312 |
| k__Bacteria;p__Proteobacteria;c__Gammaproteobacteria;o__Oceanospirillales;f__Halomonadaceae;Other | 0.0000000 | 0.0000000 |
| k__Bacteria;p__Proteobacteria;c__Gammaproteobacteria;o__Pasteurellales;f__Pasteurellaceae;Other | 0.0007018 | 0.0000000 |
| k__Bacteria;p__Proteobacteria;c__Gammaproteobacteria;o__Pasteurellales;f__Pasteurellaceae;g__Aggregatibacter | 0.0000000 | 0.0000000 |
| k__Bacteria;p__Proteobacteria;c__Gammaproteobacteria;o__Pseudomonadales;f__Moraxellaceae;g__Acinetobacter | 0.0018209 | 0.0004005 |
| k__Bacteria;p__Proteobacteria;c__Gammaproteobacteria;o__Pseudomonadales;f__Pseudomonadaceae;g__Pseudomonas | 0.0000000 | 0.0002047 |
| k__Bacteria;p__Proteobacteria;c__Gammaproteobacteria;o__Xanthomonadales;f__Xanthomonadaceae;g__Stenotrophomonas | 0.0006639 | 0.0000000 |
| k__Bacteria;p__TM7;Other;Other;Other;Other | 0.0000000 | 0.0000000 |
| k__Bacteria;p__TM7;c__TM7-3;Other;Other;Other | 0.0000379 | 0.0000000 |
| k__Bacteria;p__TM7;c__TM7-3;o__CW040;f__F16;g__ | 0.0311825 | 0.0000901 |
| k__Bacteria;p__Tenericutes;c__Mollicutes;Other;Other;Other | 0.0009484 | 0.0011865 |
| k__Bacteria;p__Tenericutes;c__Mollicutes;o__Anaeroplasmatales;f__Anaeroplasmataceae;g__Anaeroplasma | 0.5772181 | 0.8856945 |
| k__Bacteria;p__Tenericutes;c__Mollicutes;o__Mycoplasmatales;f__Mycoplasmataceae;Other | 0.0679415 | 0.0147854 |
| k__Bacteria;p__Tenericutes;c__Mollicutes;o__Mycoplasmatales;f__Mycoplasmataceae;g__ | 0.0167103 | 0.0013116 |
| k__Bacteria;p__Tenericutes;c__Mollicutes;o__RF39;f__;g__ | 0.1431285 | 0.4280979 |
| k__Bacteria;p__Thermi;c__Deinococci;o__Thermales;f__Thermaceae;g__Meiothermus | 0.0000000 | 0.0000000 |
| k__Bacteria;p__Thermi;c__Deinococci;o__Thermales;f__Thermaceae;g__Thermus | 0.0001138 | 0.0000000 |
| k__Bacteria;p__Verrucomicrobia;Other;Other;Other;Other | 0.0000000 | 0.0000686 |
| k__Bacteria;p__Verrucomicrobia;c__Verrucomicrobiae;o__Verrucomicrobiales;f__Verrucomicrobiaceae;Other | 0.0000000 | 0.0001309 |
| k__Bacteria;p__Verrucomicrobia;c__Verrucomicrobiae;o__Verrucomicrobiales;f__Verrucomicrobiaceae;g__Akkermansia | 0.0215281 | 0.1920980 |
| k__Bacteria;Other;Other;Other;Other;Other | 1.3836012 | 0.6275870 |
| Unclassified;Other;Other;Other;Other;Other | 0.0001707 | 0.0001171 |

**Supplementary table 2: Statistical analysis table.**

| **Figure** | **Panel** | **Data structure** | **Statistical test** | **Power** | **Comparison** | ***p* value** | ***N*** |
| --- | --- | --- | --- | --- | --- | --- | --- |
| 1 | D | Single variable (Treatment) | One-way ANOVA,  Tukey's multiple comparisons post hoc test | 1.0000 | *Lachnospiraceae*: All treatments | 0.0112 | Vehicle: 10 1wk ABX gvg: 12 ABX: 10 |
|  |  |  |  |  | *Lachnospiraceae*: Vehicle vs. 1wk ABX gvg | p<0.001 |  |
|  |  |  |  |  | *Lachnospiraceae*: Vehicle vs. ABX | p<0.001 |  |
|  |  |  |  |  | *Lachnospiraceae*: 1wk ABX gvg vs. ABX | p>0.05 |  |
|  |  |  |  | 1.0000 | *S24-7*: All treatments | 0.0046 |  |
|  |  |  |  |  | *S24-7*: Vehicle vs. 1wk ABX gvg | p<0.05 |  |
|  |  |  |  |  | *S24-7*: Vehicle vs. ABX | p<0.001 |  |
|  |  |  |  |  | *S24-7*: 1wk ABX gvg vs. ABX | p>0.05 |  |
|  |  |  |  | 1.0000 | *Akkermansia*: All treatments | p<0.0001 |  |
|  |  |  |  |  | *Akkermansia*: Vehicle vs. 1wk ABX gvg | p>0.05 |  |
|  |  |  |  |  | *Akkermansia*: Vehicle vs. ABX | p<0.001 |  |
|  |  |  |  |  | *Akkermansia*: 1wk ABX gvg vs. ABX | p<0.001 |  |
|  | E | Single variable (Treatment) | One-way ANOVA,  Tukey's multiple comparisons post hoc test | 0.9902 | All treatments | 0.0034 | Vehicle: 10 1wk ABX gvg: 12 ABX: 10 |
|  |  |  |  |  | Vehicle vs. 1wk ABX gvg | p>0.05 |  |
|  |  |  |  |  | Vehicle vs. ABX | p<0.05 |  |
|  |  |  |  |  | 1wk ABX gvg vs. ABX | p>0.05 |  |
| 2 | B | Simple 2 group | Un-paired two-tailed Student's *t*-test | 0.0515 | MLN: Vehicle vs. 1wk ABX gvg | 0.9044 | Vehicle: 5 1wk ABX gvg: 6 |
|  |  |  |  | 0.9364 | Blood: Vehicle vs. 1wk ABX gvg | 0.0046 |  |
|  |  |  |  | 0.5882 | Brain: Vehicle vs. 1wk ABX gvg | 0.0387 |  |
|  | C | Simple 2 group | Un-paired two-tailed Student's *t*-test | 0.2597 | MLN: Vehicle vs. 1wk ABX gvg | 0.1871 | Vehicle: 5 1wk ABX gvg: 6 |
|  |  |  |  | 0.2318 | Blood: Vehicle vs. 1wk ABX gvg | 0.2033 |  |
|  |  |  |  | 0.1169 | Brain: Vehicle vs. 1wk ABX gvg | 0.4309 |  |
| 3 | B | Simple 2 group | Un-paired two-tailed Student's *t*-test | 0.6942 | Vehicle vs. 1wk ABX gvg | 0.017 | 12 both groups |
|  | C | Simple 2 group | Un-paired two-tailed Student's *t*-test | 1.0000 | Vehicle vs. 1wk ABX gvg | 0.0105 | 12 both groups |
|  | D | Simple 2 group (2 data sets on same axis) | Un-paired two-tailed Student's *t*-test | 0.4411 | Aβ1:40: Vehicle vs. 1wk ABX gvg | 0.0712 | 14 both groups |
|  |  |  |  | 0.4047 | Aβ1:42: Vehicle vs. 1wk ABX gvg | 0.0861 |  |
|  | E | Simple 2 group (2 data sets on same axis) | Un-paired two-tailed Student's *t*-test | 0.1673 | Aβ1:40: Vehicle vs. 1wk ABX gvg | 0.314 | 14 both groups |
|  |  |  |  | 0.0671 | Aβ1:42: Vehicle vs. 1wk ABX gvg | 0.6935 |  |
|  | G | Simple 2 group | Un-paired two-tailed Student's *t*-test | 0.0767 | Vehicle vs. 1wk ABX gvg | 0.5219 | 8 both groups |
|  | H | Simple 2 group | Un-paired two-tailed Student's *t*-test | 0.0550 | Vehicle vs. 1wk ABX gvg | 0.8286 | 8 both groups |
| 4 | B | Simple 2 group | Un-paired two-tailed Student's *t*-test | 0.9985 | Vehicle vs. 1wk ABX gvg | 0.0101 | 12 both groups |
|  | C | Simple 2 group | Un-paired two-tailed Student's *t*-test | 0.0929 | Vehicle vs. 1wk ABX gvg | 0.6137 | 12 both groups |
|  | E | Simple 2 group | Un-paired two-tailed Student's *t*-test | 0.9963 | Vehicle vs. 1wk ABX gvg | 0.0102 | 12 both groups |
|  | F | Simple 2 group | Un-paired two-tailed Student's *t*-test | 0.1963 | Vehicle vs. 1wk ABX gvg | 0.3637 | 12 both groups |
|  | H | Simple 2 group | Un-paired two-tailed Student's *t*-test | 0.8379 | Vehicle vs. 1wk ABX gvg | 0.0098 | 5 both groups |
|  | I | Simple 2 group | Un-paired two-tailed Student's *t*-test | 0.7012 | Vehicle vs. 1wk ABX gvg | 0.0219 | 5 both groups |
|  | J | Simple 2 group | Un-paired two-tailed Student's *t*-test | 0.7444 | Vehicle vs. 1wk ABX gvg | 0.0174 | 5 both groups |
|  | L | Simple 2 group | Un-paired two-tailed Student's *t*-test | 0.5338 | Vehicle vs. 1wk ABX gvg | 0.0485 | 5 both groups |
|  | M | Simple 2 group | Un-paired two-tailed Student's *t*-test | 0.2639 | Vehicle vs. 1wk ABX gvg | 0.1711 | 5 both groups |
|  | N | Simple 2 group | Un-paired two-tailed Student's *t*-test | 0.4408 | Vehicle vs. 1wk ABX gvg | 0.0736 | 5 both groups |
| S1 | C | Simple 2 group (2 data sets on same axis) | Un-paired two-tailed Student's *t*-test | 1.0000 | 21 days: Vehicle vs. 1wk ABX gvg | p<0.0001 | 6 both groups |
|  |  |  |  | 0.3002 | 6.5 months: ABX vs. 1wk ABX gvg | 0.2428 | 6 both groups |
| S2 | i | Simple 2 group | Un-paired two-tailed Student's *t*-test | 0.0519 | Vehicle vs. 1wk ABX gvg | 0.8887 | Vehicle: 5 1wk ABX gvg: 6 |
|  | ii | Simple 2 group | Un-paired two-tailed Student's *t*-test | 0.2153 | Vehicle vs. 1wk ABX gvg | 0.2351 | Vehicle: 5 1wk ABX gvg: 6 |
|  | iii | Simple 2 group | Un-paired two-tailed Student's *t*-test | 0.4362 | Vehicle vs. 1wk ABX gvg | 0.0861 | Vehicle: 5 1wk ABX gvg: 6 |
|  | iv | Simple 2 group | Un-paired two-tailed Student's *t*-test | 0.2875 | Vehicle vs. 1wk ABX gvg | 0.1674 | Vehicle: 5 1wk ABX gvg: 6 |
| S3 | A | Simple 2 group | Un-paired two-tailed Student's *t*-test | 0.3097 | Vehicle vs. 1wk ABX gvg | 0.0766 | Vehicle: 12 1wk ABX gvg: 15 |
|  | B | Simple 2 group | Un-paired two-tailed Student's *t*-test | 0.1280 | Vehicle vs. 1wk ABX gvg | 0.3966 | Vehicle: 7 1wk ABX gvg: 10 |
| S4 | A | Simple 2 group | Un-paired two-tailed Student's *t*-test | 0.3271 | Vehicle vs. 1wk ABX gvg | 0.1411 | Vehicle: 5 1wk ABX gvg: 6 |
|  | B | Simple 2 group | Un-paired two-tailed Student's *t*-test | 0.0600 | Vehicle vs. 1wk ABX gvg | 0.7517 | Vehicle: 5 1wk ABX gvg: 6 |
|  | C | Simple 2 group | Un-paired two-tailed Student's *t*-test | 0.1769 | Vehicle vs. 1wk ABX gvg | 0.2637 | Vehicle: 5 1wk ABX gvg: 6 |
|  | D | Simple 2 group | Un-paired two-tailed Student's *t*-test | 0.0507 | Vehicle vs. 1wk ABX gvg | 0.9336 | Vehicle: 5 1wk ABX gvg: 6 |
|  | E | Simple 2 group | Un-paired two-tailed Student's *t*-test | 0.0528 | Vehicle vs. 1wk ABX gvg | 0.859 | Vehicle: 5 1wk ABX gvg: 6 |
|  | F | Simple 2 group | Un-paired two-tailed Student's *t*-test | N/A | Vehicle vs. 1wk ABX gvg | N/A | Vehicle: 5 1wk ABX gvg: 6 |
|  | G | Simple 2 group | Un-paired two-tailed Student's *t*-test | 0.4450 | Vehicle vs. 1wk ABX gvg | 0.067 | Vehicle: 5 1wk ABX gvg: 6 |
|  | H | Simple 2 group | Un-paired two-tailed Student's *t*-test | 0.3717 | Vehicle vs. 1wk ABX gvg | 0.088 | Vehicle: 5 1wk ABX gvg: 6 |
|  | I | Simple 2 group | Un-paired two-tailed Student's *t*-test | 0.0500 | Vehicle vs. 1wk ABX gvg | 0.9953 | Vehicle: 5 1wk ABX gvg: 6 |
|  | J | Simple 2 group | Un-paired two-tailed Student's *t*-test | 0.2268 | Vehicle vs. 1wk ABX gvg | 0.2216 | Vehicle: 5 1wk ABX gvg: 6 |
|  | K | Simple 2 group | Un-paired two-tailed Student's *t*-test | 0.0641 | Vehicle vs. 1wk ABX gvg | 0.6974 | Vehicle: 5 1wk ABX gvg: 6 |
|  | L | Simple 2 group | Un-paired two-tailed Student's *t*-test | 0.2754 | Vehicle vs. 1wk ABX gvg | 0.1502 | Vehicle: 5 1wk ABX gvg: 6 |
|  | M | Simple 2 group | Un-paired two-tailed Student's *t*-test | 0.1544 | Vehicle vs. 1wk ABX gvg | 0.3102 | Vehicle: 5 1wk ABX gvg: 6 |
|  | N | Simple 2 group | Un-paired two-tailed Student's *t*-test | 0.4380 | Vehicle vs. 1wk ABX gvg | 0.0746 | Vehicle: 5 1wk ABX gvg: 6 |
|  | O | Simple 2 group | Un-paired two-tailed Student's *t*-test | 0.0500 | Vehicle vs. 1wk ABX gvg | 0.9947 | Vehicle: 5 1wk ABX gvg: 6 |
|  | P | Simple 2 group | Un-paired two-tailed Student's *t*-test | 0.2356 | Vehicle vs. 1wk ABX gvg | 0.2044 | Vehicle: 5 1wk ABX gvg: 6 |
|  | Q | Simple 2 group | Un-paired two-tailed Student's *t*-test | 0.1583 | Vehicle vs. 1wk ABX gvg | 0.3015 | Vehicle: 5 1wk ABX gvg: 6 |
|  | R | Simple 2 group | Un-paired two-tailed Student's *t*-test | 0.0767 | Vehicle vs. 1wk ABX gvg | 0.6058 | Vehicle: 5 1wk ABX gvg: 6 |
|  | S | Simple 2 group | Un-paired two-tailed Student's *t*-test | 0.0739 | Vehicle vs. 1wk ABX gvg | 0.6328 | Vehicle: 5 1wk ABX gvg: 6 |
|  | T | Simple 2 group | Un-paired two-tailed Student's *t*-test | 0.1978 | Vehicle vs. 1wk ABX gvg | 0.2257 | Vehicle: 5 1wk ABX gvg: 6 |
|  | U | Simple 2 group | Un-paired two-tailed Student's *t*-test | 0.0800 | Vehicle vs. 1wk ABX gvg | 0.5886 | Vehicle: 5 1wk ABX gvg: 6 |
|  | V | Simple 2 group | Un-paired two-tailed Student's *t*-test | 0.1274 | Vehicle vs. 1wk ABX gvg | 0.3847 | Vehicle: 5 1wk ABX gvg: 6 |
|  | W | Simple 2 group | Un-paired two-tailed Student's *t*-test | 0.1597 | Vehicle vs. 1wk ABX gvg | 0.2908 | Vehicle: 5 1wk ABX gvg: 6 |
|  | X | Simple 2 group | Un-paired two-tailed Student's *t*-test | 0.0637 | Vehicle vs. 1wk ABX gvg | 0.703 | Vehicle: 5 1wk ABX gvg: 6 |
| S7 | A | Simple 2 group | Un-paired two-tailed Student's *t*-test | 0.0930 | Vehicle vs. 1wk ABX gvg | 0.5348 | 14 both groups |
|  | B | X/Y scatter (2 data sets on same axis) | Pearson's linear regression | 1.0000 | TFA Aβ1:40: TFA Aβ vs. 3D6 | r^2^=0.7552 | 28 both groups |
|  |  |  |  | 1.0000 | TFA Aβ1:42: TFA Aβ vs. 3D6 | r^2^=0.7876 |  |
|  | C | Simple 2 group | Un-paired two-tailed Student's *t*-test | 0.0512 | Vehicle vs. 1wk ABX gvg | 0.9175 | 14 both groups |
|  | D | Simple 2 group (2 data sets on same axis) | Un-paired two-tailed Student's *t*-test | 0.4019 | sTBS Aβ1:38: Vehicle vs. 1wk ABX gvg | 0.0874 | 11 both groups |
|  |  |  |  | N/A | sTFA Aβ1:38: Vehicle vs. 1wk ABX gvg | N/A | Vehicle: 7 1wk ABX gvg: 2 |
| S8 | B | Simple 2 group | Un-paired two-tailed Student's *t*-test | 0.0726 | Vehicle vs. 1wk ABX gvg | 0.6344 | Vehicle: 7 1wk ABX gvg: 8 |
|  | C | Simple 2 group | Un-paired two-tailed Student's *t*-test | 0.1434 | Vehicle vs. 1wk ABX gvg | 0.3559 | Vehicle: 7 1wk ABX gvg: 8 |
|  | D | Simple 2 group | Un-paired two-tailed Student's *t*-test | 0.3263 | Vehicle vs. 1wk ABX gvg | 0.1305 | Vehicle: 7 1wk ABX gvg: 8 |
|  | E | Simple 2 group | Un-paired two-tailed Student's *t*-test | 0.2241 | Vehicle vs. 1wk ABX gvg | 0.2269 | Vehicle: 7 1wk ABX gvg: 8 |
